# Trends, Advantages and Disadvantages in Combined Extracorporeal Lung and Kidney Support From a Technical Point of View

**DOI:** 10.3389/fmedt.2022.909990

**Published:** 2022-06-21

**Authors:** Ana Martins Costa, Frank Halfwerk, Bettina Wiegmann, Michael Neidlin, Jutta Arens

**Affiliations:** ^1^Engineering Organ Support Technologies Group, Department of Biomechanical Engineering, University of Twente, Enschede, Netherlands; ^2^Department of Cardiothoracic Surgery, Thorax Centrum Twente, Medisch Spectrum Twente, Enschede, Netherlands; ^3^Lower Saxony Center for Biomedical Engineering, Implant Research and Development, Hannover Medical School, Hanover, Germany; ^4^Department of Cardiothoracic, Transplantation and Vascular Surgery, Hannover Medical School, Hanover, Germany; ^5^German Center for Lung Research, BREATH, Hannover Medical School, Hanover, Germany; ^6^Department of Cardiovascular Engineering, Institute of Applied Medical Engineering, Medical Faculty, RWTH Aachen University, Aachen, Germany

**Keywords:** veno-venous ECMO, continuous RRT, extracorporeal life support, artificial kidney, artificial lung, artificial organ, kidney insufficiency

## Abstract

Extracorporeal membrane oxygenation (ECMO) provides pulmonary and/or cardiac support for critically ill patients. Due to their diseases, they are at high risk of developing acute kidney injury. In that case, continuous renal replacement therapy (CRRT) is applied to provide renal support and fluid management. The ECMO and CRRT circuits can be combined by an integrated or parallel approach. So far, all methods used for combined extracorporeal lung and kidney support present serious drawbacks. This includes not only high risks of circuit related complications such as bleeding, thrombus formation, and hemolysis, but also increase in technical workload and health care costs. In this sense, the development of a novel optimized artificial lung device with integrated renal support could offer important treatment benefits. Therefore, we conducted a review to provide technical background on existing techniques for extracorporeal lung and kidney support and give insight on important aspects to be addressed in the development of this novel highly integrated artificial lung device.

## Introduction

Extracorporeal membrane oxygenation (ECMO) is a life-saving technique used for the treatment of severe respiratory and circulatory failure refractory to conventional treatment. ECMO releases the failing lung and/or heart from its functions while it is being treated, recovered, or replaced (1). Since the development of the ECMO in the 1970s, recent technological advances in the oxygenator and pump design enables the ECMO to become a more reliable and user-friendly system (2). Patients can be supported by ECMO for days to months in three main modes: veno-arterial ECMO (VA ECMO) to provide partial-to-complete cardiopulmonary support, veno-venous ECMO (VV-ECMO) to provide partial-to-complete pulmonary support, or the combination of both as veno-veno-arterial ECMO (VVA-ECMO) in combined severe cardiopulmonary failure. Hereby, ECMO has been used for the treatment of more than 173.000 neonatal, pediatric, and adult patients worldwide. In the course of the years, especially VV-ECMO has gained attention, as an increasing number of studies confirmed an improved outcome in severe respiratory failure by ECMO treatment (3), not only in reversible respiratory failure, like acute respiratory distress syndrome (ARDS), or as bridge to lung transplantation (4, 5), also in patients suffering from COVID-19 (3, 6).

Due to high disease severity of ECMO patients and the so-called lung-kidney crosstalk, concurrent acute kidney injury (AKI) is common in up to 70% of patients and is related to significantly higher morbidity and mortality (7–9). In these cases, continuous renal replacement therapy (CRRT) is often applied and can be used in different modes including hemofiltration for fluid removal and clearance of solutes of higher molecular weights such as cytokines, hemodialysis to adjust electrolytes and urinary extracted substances; or a combination of both in hemodiafiltration where the application of a pressure gradient across the filter improves solute clearance (10).

Currently, ECMO and CRRT can be combined in two different variants improving fluid balance and electrolyte disturbances (11). First, the more common one is a parallel approach with separate vascular access for each device (11–13). This is not only associated with increased healthcare costs (14, 15) and higher technical effort [e.g., higher circuit complexity with increased technical workload (16), and circuit-related complications, but also has a significantly larger artificial surface area (17)]. Subsequently, this results in activation of the coagulation cascade and inflammation leading to thrombus formation and sepsis. Second, the integrated approach, where the RRT device is integrated in the ECMO circuit presents serious drawbacks related to controlling intra-circuit pressure differences that may lead to treatment interruption, air entrapment, flow disruption and hemolysis (16, 18). Moreover, modern ECMO devices offer connection ports specifically for integrating the CRRT to the ECMO circuit. Nevertheless, these connectors seem hemodynamically suboptimal, since blood flows deviate with a 90° angle to the CRRT access lines, which can lead to blood flow disruption, shear stress, and hemolysis.

Therefore, there is a clear need for novel extracorporeal life support technologies to consider the integration of multiple organ support, avoiding undesired side effects (12, 19) and ultimately considering the important cross-talk between lungs and kidneys (20). The combination of both extracorporeal lung and kidney support in one single device may offer relevant advantages, inter alia reduced artificial surfaces, lower numbers of required vascular accesses, which lead to reduced complications (e.g., bleeding, thrombus formation and infection), and also lower hemolysis due to the application of only one pump. Nevertheless, the development of this combined device is challenging, needing exact harmonization of different parameters like appropriate amount of and the configuration of oxygenation and hemofiltration fiber, and the type of filtration mode. Thus, this review shows trends, advantages and disadvantages of combined extracorporeal lung and kidney support from a technical viewpoint to provide insight for the development of novel devices.

## Medical Background

### Cardiopulmonary Insufficiency and ECMO

According to the Extracorporeal Life Support Organization, in the past 5 years, 81,638 patients have been supported by ECMO, among them 44% requiring pulmonary support, 42% needing cardiac support, and 14% extracorporeal cardiopulmonary resuscitation (5). Indications for ECMO are divided into three main categories depending on the organ being supported: heart, lungs, or both.

#### Indications of VA-ECMO

VA-ECMO supports patients in case of circulatory failure with or without concomitant respiratory failure. This is the case for patients with refractory shock, which can be supported by VA-ECMO while recovering from the acute incident, or patients in transition for long term-advanced therapies such as ventricular assist device or transplant. Traditional indications for VA-ECMO include acute myocardial infarction, postcardiotomy, and acute or decompensated chronic cardiac diseases. Up to 6–10% of patients that suffer from acute myocardial infraction present refractory cardiogenic shock (21–23). If treatment with less invasive methods fails, such as the administration of high doses of vasopressors, the use of VA-ECMO is considered. Therefore, VA-ECMO is indicated to avoid multiorgan failure when cardiogenic shock persists and is characterized by a low cardiac output (cardiac index of <2 L m^−1^ m^−2^), central venous O_2_ saturation <65%, lactate levels >50 mg/dL, and hypotension (systolic blood pressure of <90 mm Hg) (24). In recent years, VA-ECMO has also been considered for a number of other conditions such as preoperative ECMO, cardiocirculatory assistance during catheter based interventional cardiology, and temporary support in acute heart failure expanding the applications of ECMO for circulatory support (24).

#### Indications of VV-ECMO

VV-ECMO is indicated for the treatment of patients with any potentially reversible respiratory complication where the lungs are unable to provide sufficient ventilation and oxygenation despite the use of conventional treatment (25). The main indications of ECMO in respiratory support are divided into: (1) rescue of patients with refractory hypoxemia suffering from inadequate arterial oxygenation despite optimal levels of inspired oxygen, (2) rescue of patients with refractory hypercapnia leading to high CO_2_ blood levels, (3) rescue of patients from injurious mechanical ventilation (24). This mainly includes patients suffering from acute respiratory distress syndrome (ARDS), chronic obstructive pulmonary diseases (COPD), and waiting for a lung transplantation (26). Specific indications for the use of VV-ECMO have been reported by the Extracorporeal Life Support Organization (5, 27, 28).

#### Indications of Hybrid ECMO

Occasionally, traditional treatment with VV-ECMO or VA-ECMO is not sufficient to answer the needs of certain patients. This is the case of VV-ECMO patients requiring respiratory support that at a later stage develop cardiac decompensation, needing a VA-ECMO support. For instance, a patient with pulmonary fibrosis and pulmonary hypertension that suffers from right-heart decompensation. Moreover, patients needing VA-ECMO for cardiac support may develop severe upper-body hypoxemia requiring additional respiratory support. For these cases, a hybrid cannulation approach veno-venous arterial (VVA-ECMO) may offer a better solution (29). The use of VVA-ECMO has been reported as feasible rescue strategy for the treatment of patients suffering from combined respiratory and hemodynamic failure. Other hybrid configurations including VA-venous (VAV-ECMO) have been indicated to patients with differential hypoxia or secondary heart failure after VV ECMO initiation (30).

### Renal Insufficiency and Renal Replacement Therapy

Acute kidney injury (AKI) affects 5–7% of hospitalized patients and this incidence is even higher among critically ill patients occurring in one in four patients receiving intensive care. AKI is characterized by a severe and acute decrease in kidney function, resulting in poor elimination of fluids, creatinine, urea, and other waste products (31). If drug therapy fails to control kidney failure, AKI is typically managed by the use of treatments aiming to achieve optimal fluid balance, prevent or treat electrolyte and acid-base disturbances, and avoid further aggravation of hemodynamic and nephrotoxic injuries. Besides conventional therapies, renal replacement therapy (RRT) is the main effective technique to manage critically-ill patients with severe AKI (31, 32). The most used RRT therapies for ICU patients involve CRRT and intermittent hemodialysis (10). Both techniques require a similar extracorporeal circuit, however mainly differ on the duration of the therapy and type of solute clearance (31, 33). The use of CRRT is preferred over IHD for hemodynamically unstable patients with AKI and patients with brain injury, in which important fluctuations in solute concentration and fluid volumes are prejudicial (34). Numerous studies evaluated differences in outcome between intermittent and continuous renal replacement modalities, however to date neither technique has shown to be superior in terms of mortality (35–37).

The choice of RRT modality appears to be strongly related to physician preferences, organization characteristics, and patient characteristics such as age, severity of illness and comorbidities (38). CRRT is typically applied to deliver both, solute clearance and fluid removal, in the form of continuous veno-venous hemofiltration (CVVH), continuous veno-venous hemodialysis (CVVHD), or continuous veno-venous hemodiafiltration (CVVHDF). The different mechanism of solute clearance between these modalities are presented in section The Hemodiafilter. Clinicians can select different CRRT modalities depending on the patient demand on solute clearance and removal. Further details on clinical applications of specific CRRT modalities have been described by multiple authors (10, 39–42).

#### Lung-Kidney Crosstalk and Combined Insufficiency

**Figure 1:**
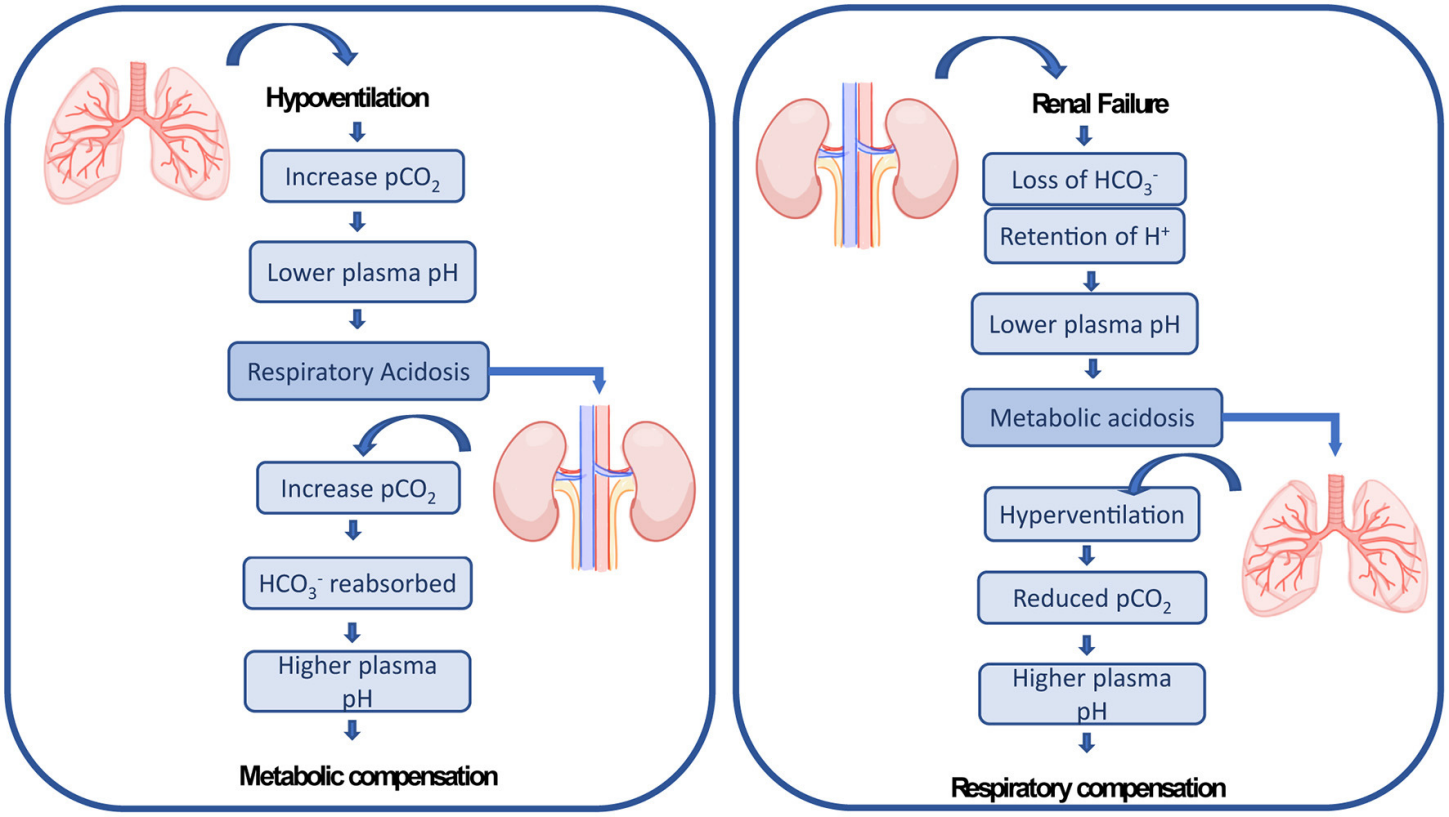
The lungs and the kidneys cross-talk mechanism of renal compensation to pulmonary acidosis and pulmonary compensation to metabolic acidosis as explained by Sorino et al. (20).

ECMO patients typically present multiple hemodynamic, inflammatory and pathophysiologic abnormalities resulting from existing comorbidities and exposure to the ECMO circuit (13, 24), which puts them at risk of other organ failures. Acute kidney injury (AKI) with fluid overload are common complications affecting ECMO patients. The administration of large volumes of intravenous fluid is required after ECMO is initiated to establish an appropriate ECMO flow, treat hemorrhage, or to minimize venous access insufficiency (27, 43). These fluid fluctuations affect the capacity of the kidney to maintain fluid hemostasis compromising the organ (44). AKI affects up to 70% of ECMO patients (11, 45) and is characterized by an abrupt loss in renal excretory function comprising both structural organ damage and function impairment, resulting in increased mortality (46–48). There are multiple causes leading to AKI in ECMO patients including preexisting comorbidities factors, such as acute inflammatory and immune-mediated processes, the use of nephrotoxic medication, which can be aggravated due to the lung-kidney crosstalk (20). The lungs and the kidneys work together to maintain the acid-base balance in the blood. Therefore, by a crosstalk mechanism, respiratory dysregulation is compensated by metabolic adaptation and the other way around (see Figure 1). Combined pulmonary-renal complications are frequent, since initial impairment of the lungs leads to dysregulation of the kidneys and vice-versa (20). Therefore, AKI is reported to be present before or after ECMO has been established (16, 47, 49, 50).

#### Indications of RRT for Combined Lung and Kidney Insufficiency

The treatment and prevention of fluid overload and AKI in ECMO patients is typically managed using renal replacement therapy (RRT). Studies show that between 20 and 100% of ECMO patients suffering from AKI are treated with RRT before or during the ECMO treatment (11, 51, 52). CRRT is the most common modality used in the treatment of ECMO patients. In a survey conducted between 65 international ECMO centers, CRRT was mainly indicated to treat fluid overload (43%), prevent fluid overload (16%), treat AKI (35%), and control electrolyte disturbances (4%) (47). The ECMO and CRRT were mainly combined by an integration of an in-line hemofilter (21.5%) or a CRRT device to the ECMO (50.8%) circuit, see section Combined Pulmonary and Renal Support. Therapy combining ECMO and CRRT has been applied to patients of all ages. Nevertheless, providing ECMO and CRRT via independent circuits has been recommended for adult patients (53). So far guidelines on the timing to start the renal support remains unclear (12, 13).

## Technical Aspects

### The Extracorporeal Membrane Oxygenation Circuit

**Figure 2:**
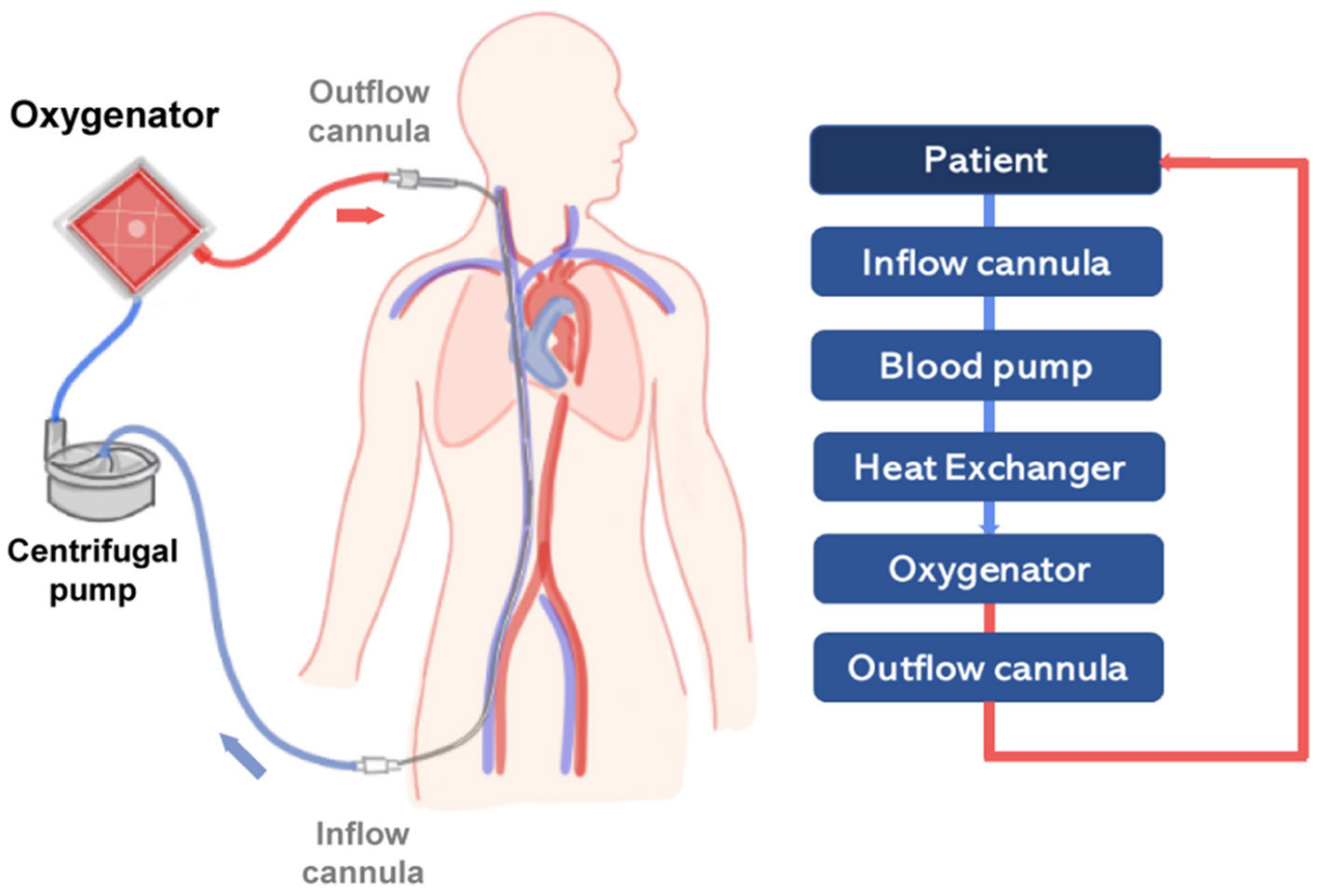
Scheme representing a simplified VV-ECMO circuit, providing partial to complete pulmonary support in severe respiratory failure. On the right side, a diagram represents the main components of the circuit (light blue boxes) connected to the patient (dark blue box). The flow of oxygenated blood is represented by a red line and deoxygenated blood by a blue line.

In general, the ECMO circuit consists of the oxygenator, the pump, and additional components such as cannulas, tubing and a heat exchanger. Blood is first removed from a large central vein of the patient, typically the jugular or femoral vein, and pumped across the gas exchange device known as oxygenator (see Figure 2). Vascular access is obtained using cannulas, which are special catheters inserted directly into the vessel. Traditionally, two cannulas of sizes ranging from 21 to 28 Fr size are used in adult VV-ECMO. However, modern dual-lumen catheters can also be used, providing the advantage of needing a single access vein and reducing the incidence of vascular trauma (54). In detail, venous blood is transported across the oxygenator with the aid of a rotary blood pump that transports fluid from access to the return point in the circuit. The design of modern centrifugal pumps allows for prolonged perfusion, reduced blood stagnation and thrombosis, and low heat production (24).

After passing through the centrifugal pump, the blood flow is directed to the oxygenator. The core of actual oxygenators is composed of thousands of small hollow fiber membranes with an outer diameter of 380 μm (55, 56). These gas exchange membranes, similarly to the lung alveoli, provide the necessary interface for O_2_-application and CO_2_-removal, to reinfuse oxygenated and decarboxylated blood into the patient. In VA-ECMO blood is removed and returned directly to the aorta commonly *via* the femoral artery. The VA-ECMO circuit bypasses both lungs and heart providing cardiac and respiratory support (57). VV-ECMO relies on the patient's own cardiac function.

Tubing is used in the circuit to transport blood, connecting the patient and the main ECMO components. Tubes are typically made of polyvinylchloride, polyurethane or silicone rubber and receive coating to reduce systemic inflammatory response and risk of blood clotting. Examples of coatings include albumin, phosphorylcholine and heparin (57). The blood temperature is maintained by circulating tempered water through a compact and efficient heat exchanger, which is typically integrated to the oxygenator.

#### The Oxygenator

The core of most gas exchange devices is composed of plasma tight hollow fiber PMP membranes with a surface area up to 2.5 m^2^ in adult devices. PMP membranes allow high gas permeation as their predecessor's polypropylene fibers, however, are produced with a compressed outer layer that prevents plasma from leaking to the fiber lumen (24). In modern oxygenators, blood flows outside of the hollow fiber membranes while gas is transported through the lumen of the hollow fibers. Gas transported across the oxygenator is referred to as sweep gas. In most cases, sweep gas is composed of pure oxygen. Occasionally, the composition of the sweep gas needs to be tuned by the mixing of oxygen with other gases and in such cases an air-blender is used. The gas exchange capacity of oxygenators is influenced by the configuration of hollow fiber membranes (2, 58). Oxygenation fibers are produced with a defined configuration in the form of mats composed of knitted hollow fiber membranes of 380 μm in diameter spaced evenly within the same layer (56). Thousands of those arranged fibers are placed in the oxygenator. Typically, the hollow fiber membranes as well as the surface of the complete ECMO device and heat exchanger are coated to increase hemocompatibility and reduce thrombus formation. Different substances have been applied for coatings such as heparin/albumin and phosphorylcoline (24).

The design of ECMO devices has evolved to implement special features, sensors and connectors between the gas exchange device and other organ support therapies (59).

#### Gas Exchange in the Oxygenator

**Figure 3:**
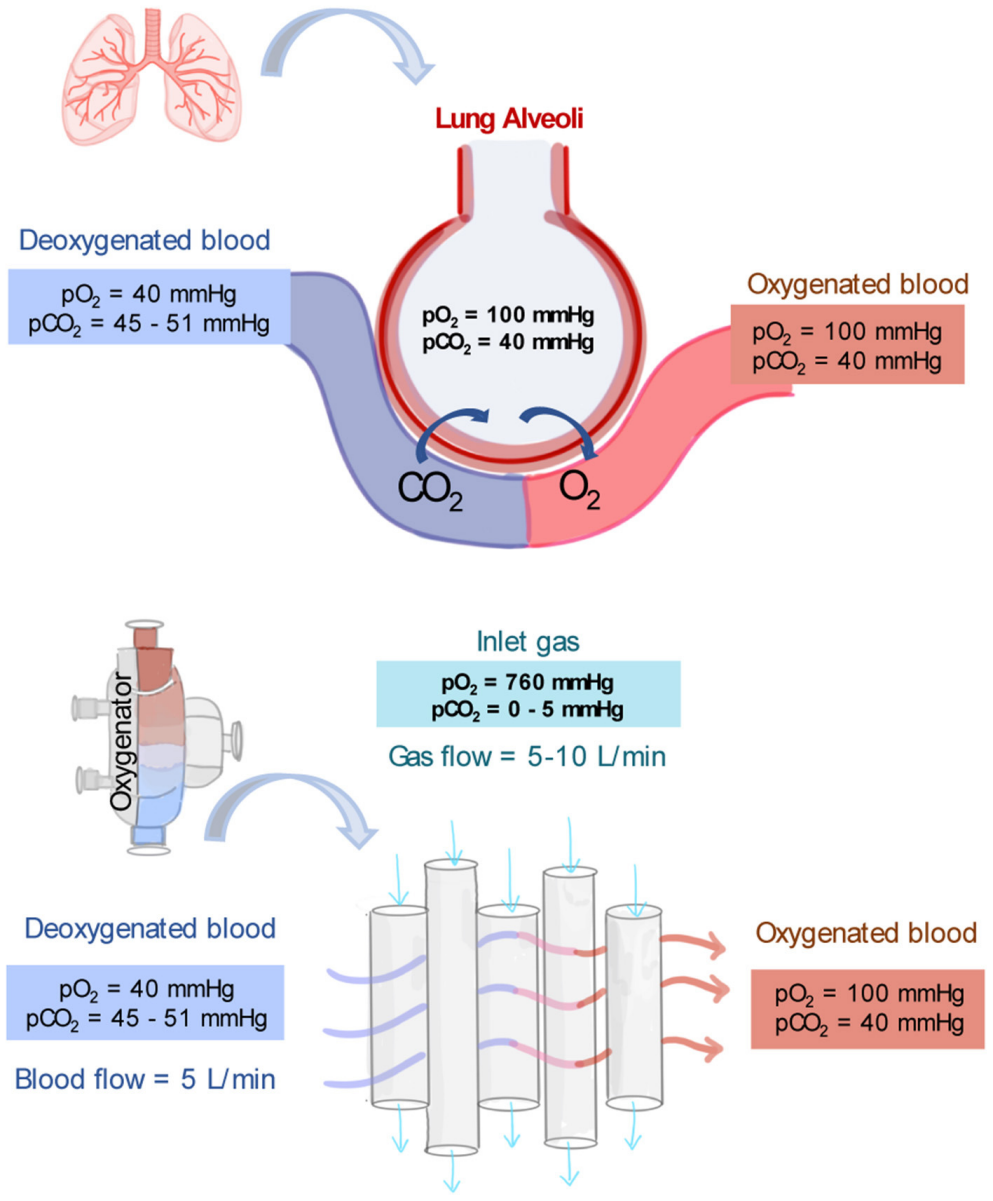
Typical partial pressures of oxygen (pO_2_) and carbon dioxide (pCO_2_) in the lung's alveoli, in deoxygenated blood and oxygenated blood. In addition, typical operating conditions and partial pressure of gases as driving force for gas exchange in the core of oxygenators (55, 56).

An oxygenator's typical operating conditions are summarized in Figure 3. Deoxygenated blood collected from the patient venous system enters the oxygenator with a typical pO_2_ of 40 mmHg and a pCO_2_ of 45–50 mmHg (56). This venous blood passes outside of the hollow fibers and the exchange of gases takes place at the membrane-blood interface. The amount of oxygen delivered by the oxygenator depends on the membrane surface area, the amount of blood mixing, the blood flow rate, and the oxygen carrying capacity of the blood (which depends on the content of hemoglobin and the saturation of blood at the inlet and outlet). After passing through the oxygenator, blood fully saturated in oxygen returns to the patient. A blood oxygenator providing complete respiratory function, as in VV-ECMO, must transfer up to 250 ml/min of oxygen to meet the minimal respiratory requirement of an adult patient (60). Technical strategies are used so that blood oxygenators can provide the basal metabolic amount of oxygen to the blood as would occur in the lungs. First, in an oxygenator, the blood is exposed to higher partial pressure gradients of O_2_ and CO_2_ than in the lung's alveoli. The feed gas flowing through the bore side of the membranes is enriched in oxygen or pure oxygen with an oxygen partial pressure (pO_2_) of 760 mmHg (55). Therefore, the sweep gas has a higher pO_2_ and lower partial pressure of CO_2_ (pCO_2_) than what found in alveolar air and driving forces up to 500–600 mmHg can be reached. The second strategy is that sweep gas is constantly flowing through the lumen of the membrane fiber, while in the lungs, oxygen enters the alveolus in breathing cycles.

In parallel, the blood oxygenator device must remove about 200 ml/min of carbon dioxide from the body. Since the ratio of CO_2_ production to O_2_ metabolism by tissues and organs is about 0.8, the same CO_2_-to-O_2_ ratio must be met in an artificial lung device to avoid hypercapnia (abnormally elevated pCO_2_ in the blood) or hypocapnia (abnormally low pCO_2_ in the blood). The removal of CO_2_ is controlled by the gradient of pCO_2_ in the venous blood and in the sweep gas (which is usually zero). The higher the gas flow rate, the lower the concentration of carbon dioxide in the sweep gas, and the higher the driving force for carbon dioxide elimination (60). CO_2_ has a higher solubility and diffusivity than O_2_ therefore its removal is typically more efficient. Thus, while effective CO_2_ elimination could be achieved with blood flows as low as 10–15 ml/kg/min, effective oxygenation requires much higher flows up to 80–100 ml/kg/min (57).

### Renal Replacement Therapy

#### Renal Replacement Therapy Circuit

**Figure 4:**
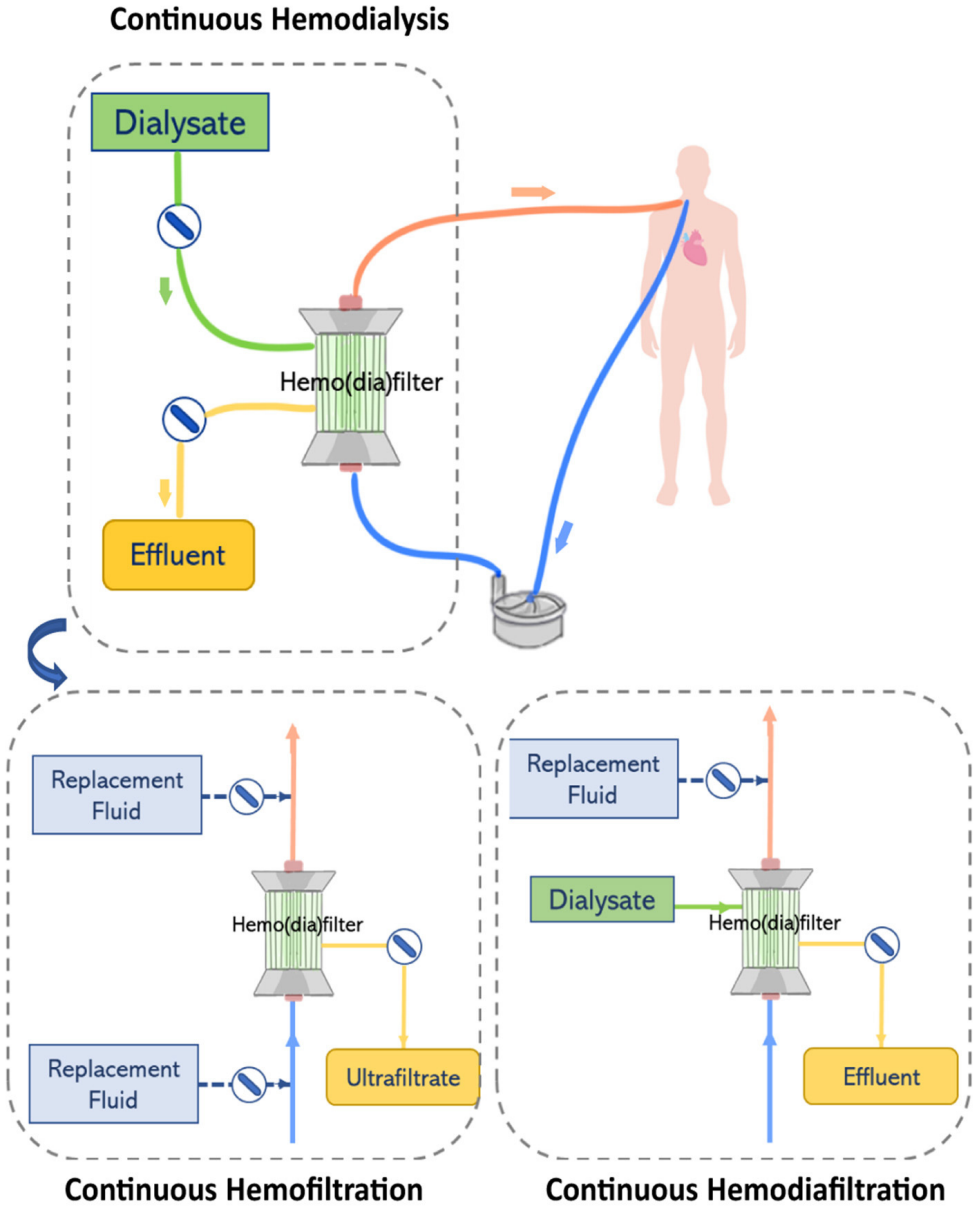
The renal replacement therapy circuit while providing continuous hemodialysis, continuous hemofiltration, and continuous hemodiafiltration. Replacement fluid can be delivered before or after the hemofilter.

**Figure 5:**
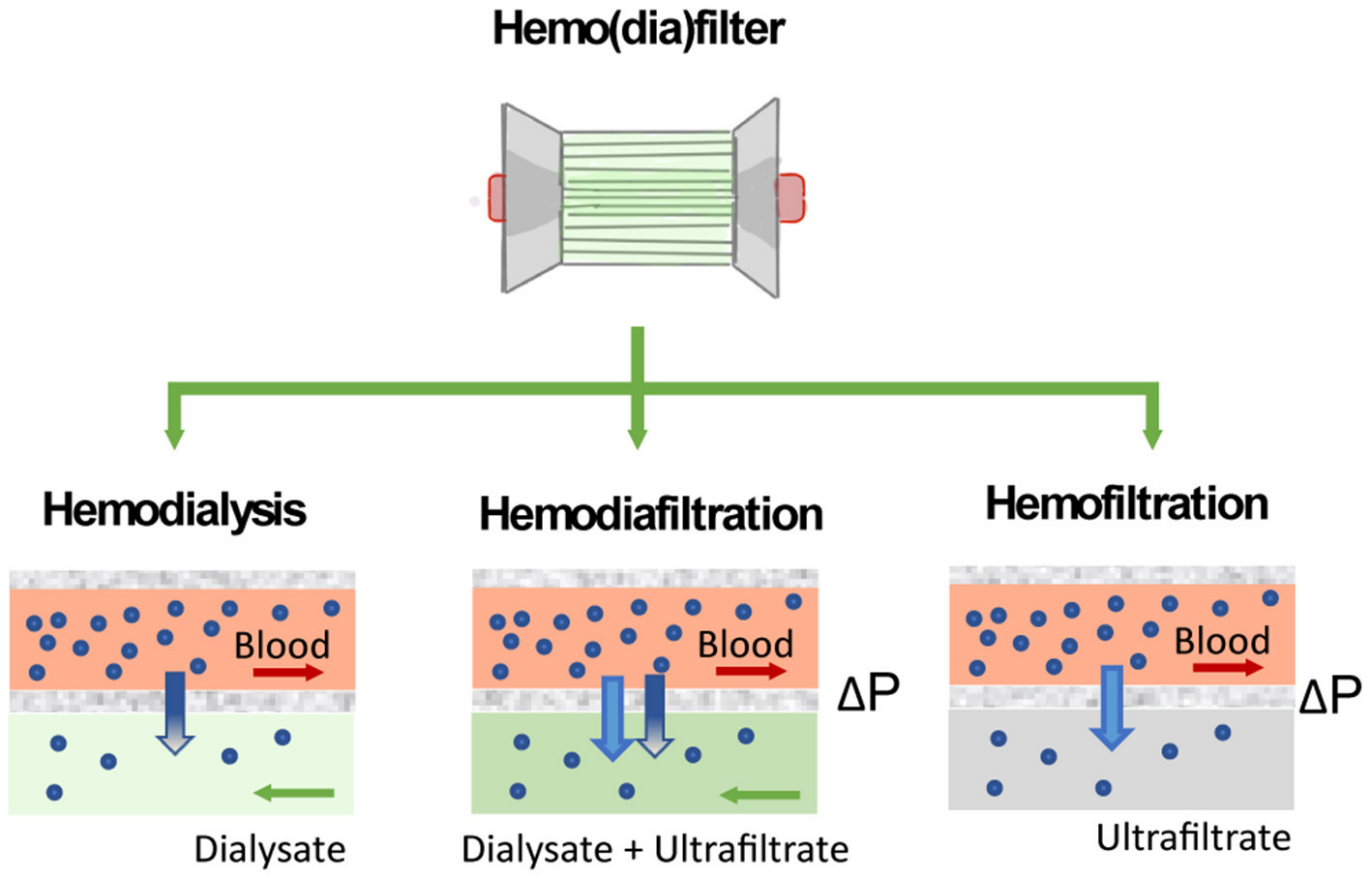
Solute clearance in renal replacement therapy can be achieved by diffusion or convection. In diffusion-based methods (hemodialysis), solutes move down their concentration gradient from the bloodstream to a dialysate solution. Methods based on convection (hemofiltration) apply a pressure gradient (ΔP) to force solutes and fluid flow across the filter.

Renal replacement therapy is delivered by an extracorporeal circuit, which can be divided into an inflow segment, a blood pump, a hemofilter and an outflow segment (44) (see Figure 4). Access to blood circulation is typically provided by a double lumen catheter placed in a location of high blood flow usually into the right internal jugular vein or in femoral veins (61). A blood pump is required to control blood flow across the circuit. Typically, roller pumps are chosen since they are less expensive than centrifugal pumps, however, these pumps can cause blood damage and have limited times of usage (44). The pump circulates blood through the hemofilter where solutes and fluids are exchanged by diffusion and/or convection (Figure 5). Depending on the chosen RRT modality, the delivery of blood and dialysate to the hemofilter can differ.

In CVVHD, blood flows through the hemodialyzer in one direction, while dialysate perfuses the hemodialyzer on the opposite side and flows in countercurrent to the blood flow direction. Solutes clearance is achieved by diffusion. A pump is used to control the flow of dialysate through the hemofilter and maintain differences in concentration gradients that drive the transport of solutes (44). In contrast, in CVVH, blood flowing through the hemofilter is submitted to a pressure gradient that leads to the formation of ultrafiltrate. Excess ultrafiltrate is compensated by the infusion of replacement fluid to achieve fluid balance. Two infusion pumps are used to control the delivery of replacement fluid to the circuit either before and/or after the hemofilter. High ultrafiltration rates used in CVVH concentrate the blood while it passes through the hemofilter. Therefore, infusion of replacement fluids before the hemofilter dilutes the blood entering the filter balancing the concentration effect. However, the delivery of a replacement fluid also dilutes the solutes in the blood, reducing the driving force in concentration gradients and consequently lowering solute clearance at a defined ultrafiltration rate (10). Thus, this effect can be reduced by infusing replacement fluids after the hemofilter. In CVVHDF, like hemodialysis, dialysate is perfused on the opposite side of the hemodiafilter and flows in countercurrent to the direction of the blood flow. However, in this case, the effluent leaving the hemodiafilter consists of dialysate plus ultrafiltrate volume. Therefore, a pump is used to deliver replacement solution before or after the hemodiafilter (44).

#### The Hemodiafilter

Modern hemo(dia)filters are made of numerous hollow fiber membranes composed of synthetic polymers such as polysulfone (PSu) or polyethersulfone (PES), which are organized in a fiber bundle configuration. In the hemo(dia)filter, blood is transported through the lumen of such hollow fiber while dialysate or ultrafiltrate flow outside of the fibers. These hollow fiber membranes typically present an asymmetric structure with a thin dense layer on the lumen side that determines their sieving coefficient and solute clearance (62). Commonly used hemo(dia)filtration hollow fiber membranes have a mean molecular permeability of ~30 kDa and high flux water permeability >25 ml/h/mmHg/m^2^. The total number of hollow fiber membranes inside the hemodiafilter accounts for a total surface of ~0.6–2 m^2^ (44).

The performance of the hemo(dia)filter depends on its capacity to eliminate blood toxins and excessive fluid from the patients. Concerning toxins elimination, solutes in the blood are mainly classified by their molecular weight (63). Molecules of small molecular weight (<500 Da) such as glucose, lipids, urea, electrolytes, and creatinine are eliminated by a healthy kidney through ion channels or diffusion through the cell membrane. Other molecules such as hemoglobulin and bilirubin, which have molecular weight ranging from 500 to 15 kDa are metabolized by different pathways in the human body. Finally, molecules of large molecular weight (>15 kDa) are enable to permeate the membrane wall of most human cells (64). In the hemo(dia)filter solute clearance can be tuned by applying different modalities (Figure 5). The semipermeable hollow fiber act as a contactor between the blood and filtrating solution or dialysate and solutes can be eliminated in different ways depending on driving forces across the membrane. In CVVH, solutes are transported by convection driven by a transmembrane pressure that creates a high ultrafiltration rate across the membrane. Solutes are entrained and dragged by the fluid flow permeating the membrane in a process typically referred to as solvent drag (65). In contrast, no transmembrane pressure is applied in CVVHD. Dialysate flows outside of the surface of dialysis membranes allowing solutes to diffuse from blood to dialysate driven by their concentration gradient (10, 62). CVVHDF is a combination of both techniques, which applies a pressure gradient across the membrane to improve the elimination of solutes and filtration performance.

Different commercial CRRT devices are applied in clinics to provide solute clearance and fluid balance. The latest generation of CRRT devices including the PrisMax (Baxter), the MultiFiltrate Pro (Fresenius), and the Omni (Braun) focus on innovations to reduce device complexity for users while delivering target prescriptions, tune pump speeds to achieve desired solute clearance, and simplify handling of fluid replacement systems. These innovations aim to improve device efficiency, pump abilities, and expand the type of purification therapies. The CRRT device contains various sensors to monitor pressures at different zones of the circuit. Moreover, safety features are typically included to minimize complications including a bubble trap and gas detector to prevent air embolism (44). Nevertheless, future developments including compatibility with pediatric filters and ECMO are yet to be introduced (66).

## Combined Pulmonary and Renal Support

The combination of ECMO and CRRT is frequently applied to control fluid balance and electrolyte levels in ECMO patients with AKI (11). Controlling fluid overload by CRRT early in the treatment may be associated with improved outcomes. However, there is a wide variation on modalities used by clinical centers to combine ECMO and CRRT devices (47). During ECMO, CRRT can be provided by two main approaches: an integrated approach or by parallel systems. In the integrated approach, a hemofilter or CRRT machine is introduced into the ECMO circuit. This is the most used method to combine ECMO and CRRT devices which has the advantage to be comparatively simple and inexpensive (67). Moreover smaller priming volumes are required when compared to the use of two independent circuits. On the other hand, in the parallel approach, the CRRT and ECMO circuits can work in separate circuits *via* independent catheters (11, 18). Different RRT modalities including continuous veno-venous haemodialysis, continuous veno-venous haemofiltration, continuous veno-venous haemodiafiltration and slow continuous ultrafiltration can be delivered using any of the two connection approaches (13). Nevertheless, the combination of CRRT and ECMO is challenging, especially since it may require additional vascular access points and expert knowledge to manage pressure levels in the circuits (18). So far, there are no clear recommendations on which particular connection between ECMO and CRRT should be used or optimal approach to combine them. Existing techniques present each their advantages and drawbacks, see Table 1, however, all of them make use of two circuits to provide lung and kidney support in critically-ill patients.

**Table 1:** Review of main available methods of combining continuous renal replacement therapy (CRRT) and extracorporeal membrane oxygenation (ECMO) (11, 12, 18).

**Combination of CRRT and ECMO**	**Advantages**	**Drawbacks**
In-line Hemofilter	• Relatively simple and inexpensive • Requires a smaller priming volume compared with the use of a separate CRRT circuit	• Requires external infusion pumps to control ultrafiltration
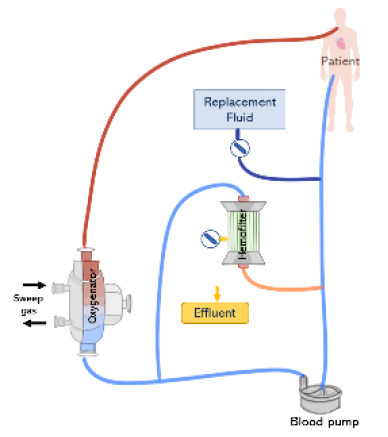		• Difficult to control volume of fluids delivered to patient
		• May increase technical workload to control fluid delivery
		• Flow disturbances
		• Limited solute clearance
Integration of CRRT in the ECMO circuit before the pump	• No additional vascular access • Enables the display of pressures • Better control of ultrafiltration • External pumps are not necessary	• Intra-circuit pressure changes may be outside the safety range of the CRRT machine
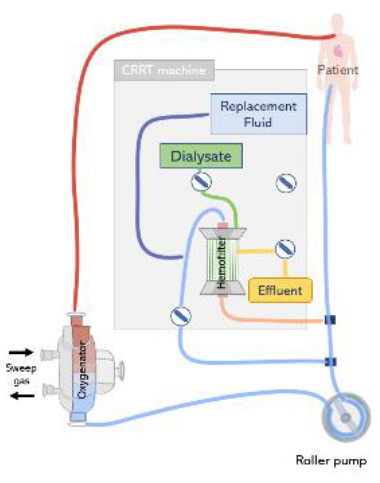		• Pressure alarm trigger multiple stops of the CRRT device • Risk of clotting • Risk of air entrapment
Integration of CRRT in the ECMO circuit after the pump	• Reduces risks of air entrapment when centrifugal pump is used	• Positive pressure triggers high pressure alarm in the access line of the CRRT
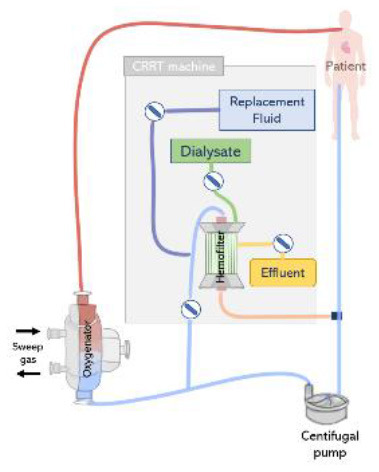	• Good inflow to the CRRT inlet • Low resistance to receive CRRT outlet	• Triggers low pressure alarm in the outlet line of the CRRT • Iterative stops in the treatment • Risk of clotting
Combination of CRRT with ECMO via existing Luer locks	• Pressure range is tolerated by the CRRT machines	• Connection risks to interfere with the oxygenator
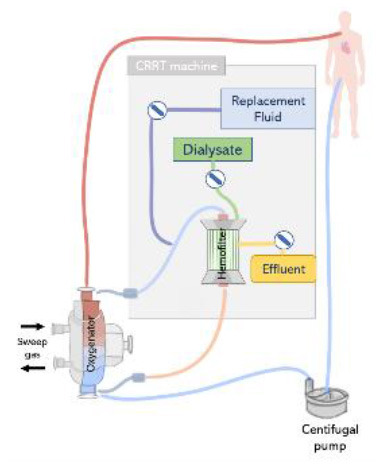	• Ultrafiltration can be regulated • Oxygenator prevents air entrapment	• Pressure may be high for acces and return CRRT lines • Pressure alarms stop the treatment blocking blood flow
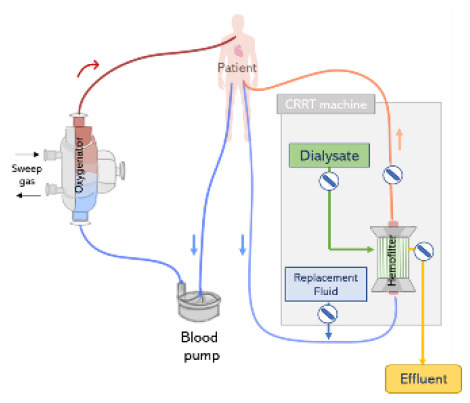	• Eminates potential interferences between both extracorporeal techniques • Fluid removal is controlled	• Requires additional cannulation • Higher risks of bleeding and infection • Increases system complexity • Higher priming volume

### Parallel Application

**Figure 6:**
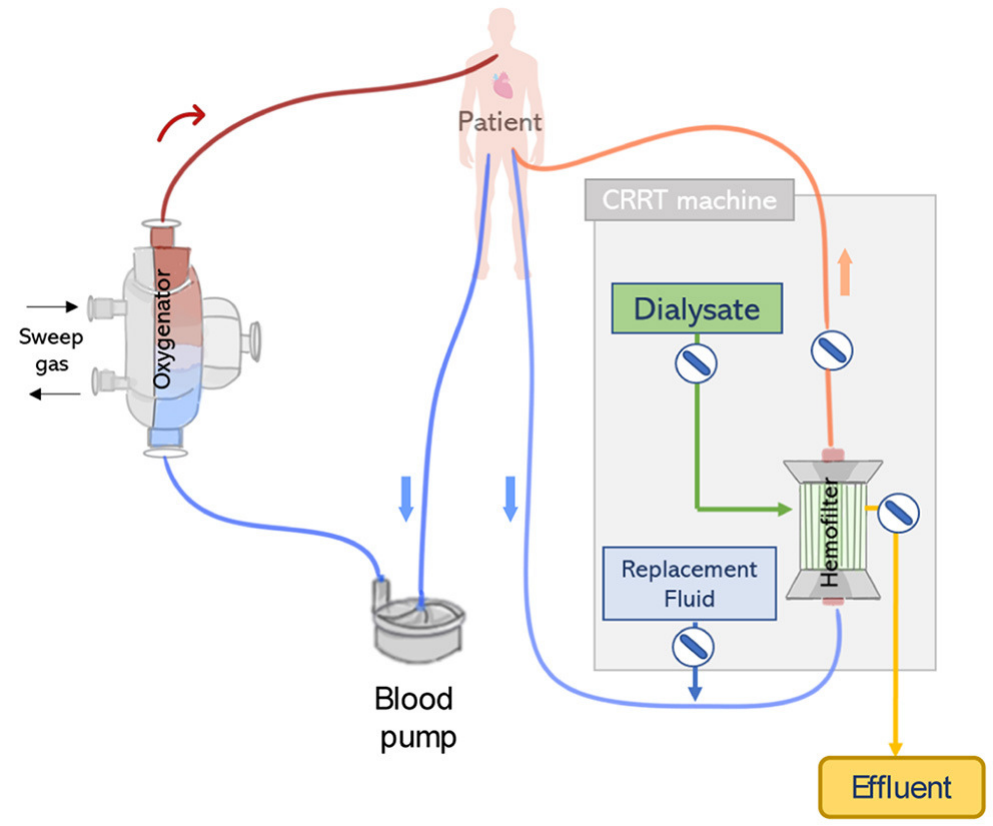
CRRT and extracorporeal membrane oxygenation ECMO connected to the patient by two independent circuits. The ECMO and CRRT machines are connected to the patient via separate vascular accesses.

The simplest and one of the most frequent forms to connect ECMO and CRRT is via independent venous access and circuits (see Figure 6). Considering that flows within the ECMO circuit (typically 4,000–5,000 ml/min) are much higher than those used in the CRRT circuit (~150–200 ml/min), a clear advantage of running ECMO and CRRT in independent circuits is that possible interferences between circuits and concerns regarding pressure alarms are eliminated. Therefore, a standard procedure can be applied for managing the CRRT circuit, including the change of the hemo(dia)filter, without requiring direct involvement of the ECMO perfusion specialist. Moreover, anticoagulation can be delivered in a combined approach (systemic and regional). Studies defend the use of parallel ECMO and CRRT circuits as a preferred treatment for adult patients. This is due to important drawbacks related to the integration of a CRRT device into the ECMO circuit which in summary results in dramatic differences in flow and pressure, increasing shear stress and hemolysis, exposing the patient to severe risks such as intravascular coagulation and systemic inflammation (68, 69), see section Integrated Application. Due to these disadvantages, Jacobs et al. strongly argued against the use of combined ECMO and CRRT in an integrated approach. The authors advocate that an independent CRRT would be a better solution which can run under a specific anticoagulation therapy (e.g., regional citrate) avoiding the dilution of anticoagulants by the ECMO system and consequently reducing thrombotic events (53).

Nevertheless, the use of ECMO and CRRT *via* independent circuits presents a number of disadvantages. First, in this configuration, separate vascular access is required, therefore an extra venous catheter is necessary to deliver CRRT in addition to the two cannulas typically required for ECMO. Thus, this is a complex requirement for patients who may not have additional cannulation sites available. Moreover, additional vascular access is associated with complications such as bleeding (especially since patients typically receive high doses of anticoagulants), infection, and thrombosis (13, 70). In addition, the use of two separate circuits accounts for large artificial surfaces, which is related to complications such as activation of the coagulation cascade and thrombus formation. Moreover, the complexity of the complete circuit increases since additional blood pumps are used, which is in turn related to increased shear stress and hemolysis (16).

### Integrated Application

#### Introduction of an In-line Hemofilter Into the ECMO Circuit

**Figure 7:**
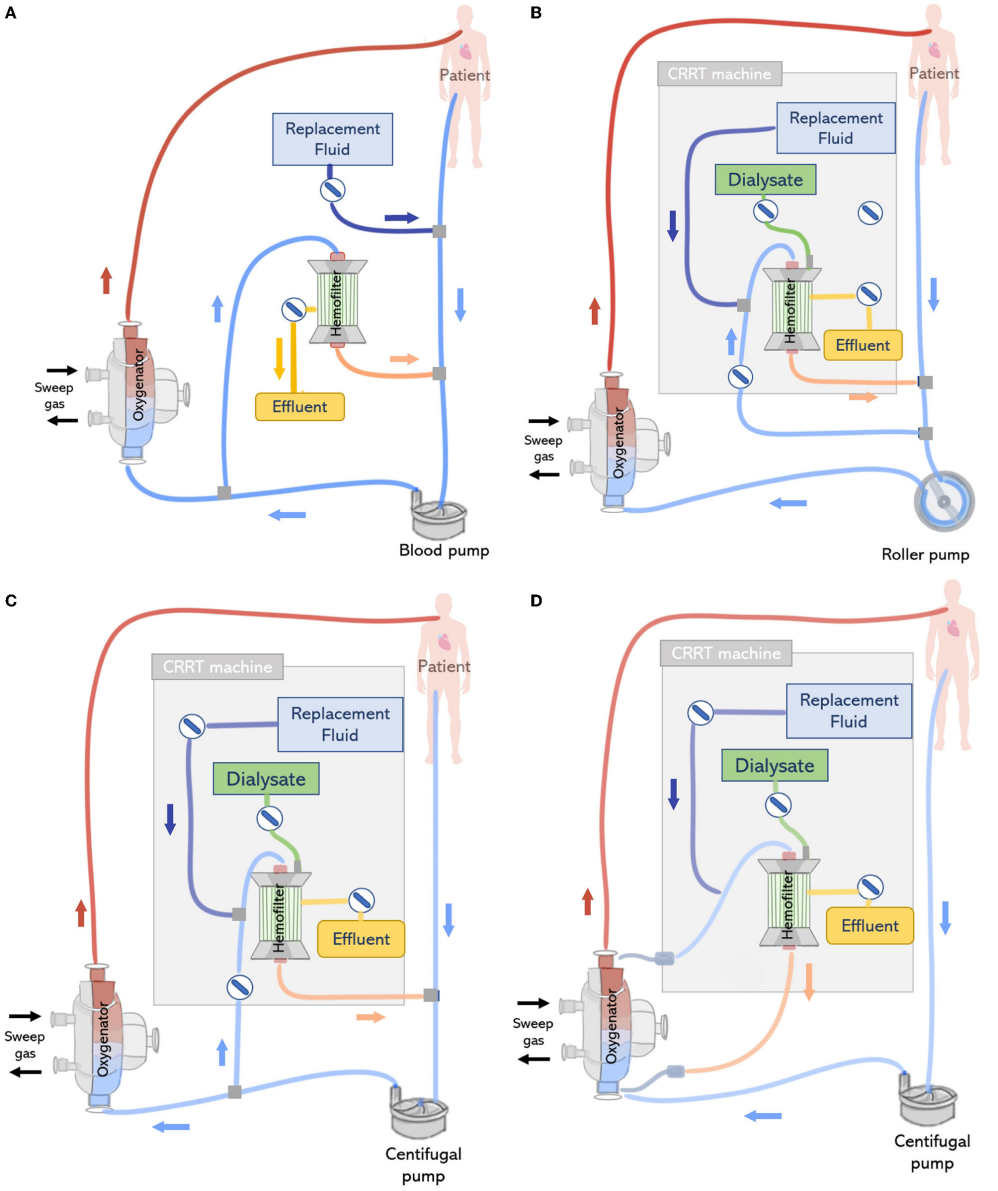
(Top left) **(A)** In-line connection of a hemofilter to the ECMO circuit. The hemofilter is connected to the high-pressure part of the ECMO circuit after the blood pump or post oxygenator. (Top right) **(B)** Connection of a CRRT machine to the ECMO circuit with a roller pump. The inlet of the CRRT circuit is connected to the ECMO circuit before the pump and CRRT outlet is returned before the pump and ECMO bladder or directly to the bladder. (Bottom left) **(C)** Connection of a CRRT machine to the ECMO circuit with a centrifugal pump. The inlet access of the CRRT machine is connected to the high-pressure line of the ECMO circuit and the CRRT outlet is returned before the pump. (Bottom right) **(D)** Connecting the CRRT machine to the ECMO circuit via existing access points pre-oxygenator and post-oxygenator.

Renal support during ECMO can be provided by placing a hemofilter in-line to the ECMO circuit (Figure 7A). A positive pressure is typically required to forward the blood flow through the filter, which is therefore connected after the pump. The outlet of the hemofilter is generally reconnected after the pump and before the oxygenator allowing purified blood to return to the ECMO circuit, using the oxygenator as air and clot entrapment. The blood flow rate passing through the hemofilter corresponds to the difference between the total ECMO blood flow rate and the actual flow rate being delivered to the patient (71). Since a tube ramification exists before the pump, the blood flow measured by the pump and the actual flow delivered to the patient may be different. Therefore, the volume of effluent and replacement fluid is controlled by two infusion pumps, one connected to the outflow of the hemofilter and the other before to the drainage line or bladder (12). One challenge of this approach consists in determining the amount of fluid being delivered to the patient. Several methods have been developed for this purpose (11, 12). One of the methods is to assume that the amount of fluid removed or delivered is equal to the flow rate of the infusion pumps, however, this is related to inaccuracies. As an example, Sucosky et al. reported standard errors in net ultrafiltrate volume removed from a patient were reported to be as high as 845 ± 156 ml over a period of 24 h (72). Differences between observed and actual fluid removal have been related to patient dehydration. An alternative more precise approach consists of weighting or measuring the real volume of ultrafiltrate removed (11). However, this requires nursing staff to control the amount of fluid removed, increasing their workload (67). Another possibility is to adjust the blood flow rate in the haemofilter using flow-restricting connectors such as a stopcock, however this is related to blood flow disturbances, risk of hemolysis and blood coagulation (71). In addition, hemofilters are designed to work with low pressure system and the maximum volume of fluid delivered by the infusion pump is limited to 1 L/h. Therefore, convective and diffusive clearance are limited with the hemofilter when compared to conventional CRRT membranes. Typically, the in-line hemofilter method is applied when slow continuous ultrafiltration needs to be delivered (47).

#### Introduction of a CRRT Device Into the ECMO Circuit

ECMO circuits may offer the option to support additional organ supporting therapies, including CRRT. The connection of an external CRRT machine to the ECMO circuit is reported is one of the preferred approaches for providing of continuous veno-venous hemofiltration, continuous veno-venous hemodiafiltration, and continuous veno-venous hemodialysis (47). This offers the advantages of a better control of effluent without the help of external pumps and precise monitoring of transmembrane pressure. However, working pressures in the ECMO and CRRT circuit differ, therefore, this approach is often associated with issues regarding the CRRT machine pressure alarms in the inlet and outlet connection lines (12).

The CRRT machine is typically connected to the venous line of the ECMO circuit, however, depending on the type of ECMO pump used (roller pump or centrifugal pump), CRRT inlet can be located before or after the pump (73). If a roller pump is used, it is possible to connect the CRRT machine to the ECMO circuit before the pump, in a way that blood is driven from the ECMO circuit to the CRRT device, circulated through the renal supporting device and returns to the circuit before the pump (Figure 7B). However, in circuits containing a centrifugal pump, the CRRT device must be placed after the pump to avoid air entrapment (12) (Figure 7C). In any case, higher blood pump rotations may generate very low blood pressures, resulting in risks specially when the inflow to the ECMO circuit is limited (71). Moreover, it is essential that blood leaving the CRRT be returned before the oxygenator to trap air, blood clots and prevent venous admixture due to the tube ramification (67).

In addition, depending on the CRRT machine and its position in the ECMO circuit, the introduction of a CRRT device may lead to intra-circuit pressure variations which could trigger pressure alarms. Blood pressures in the ECMO circuit are negative before the blood pump ranging from −20 to −100 mmHg and positive in the zone between the pump and the oxygenator with a range of +150 mmHg to +350 mmHg. On the other hand, commercial CRRT machines are manufactured to work with venous pressures between 0 and 20 mmHg. If pressures are beyond the expected range, CRRT machines are equipped with pressure alarms in the access/arterial line (−250 mmHg; +250 mmHg) and return/venous line (−50 mmHg; +350 mmHg) to warn the user (18). For instance, the arterial alarm pressure of the CRRT device may be offset if blood leaving the CRRT device is returned to the ECMO circuit before the pump, due to low inlet pressures in the negative pressure section of the ECMO circuit (−20 to −100 mmHg). Some commercial CRRT machines can recognize that these changes in pressure are related to the ECMO connection, however, in other cases, this may lead to interruptions in the treatment due to iterative stops, resulting in complications such as air entrapment and flow disturbances (13). In the clinical setting, this is generally countered by de-activating pressure alarms or adjusting ECMO blood flows, with the use of clamps and pressure control lines. However, these practices may lead to turbulence and hemolysis, and their safety is yet to be completely evaluated (18). Moreover, overriding pressure limits leads to low negative pressures, resulting in hemolysis and microembolization (12). In a third configuration, CRRT can be directly connected to the inlet and outlet ports of the oxygenator, which are used for priming and monitoring the transmembrane pressure (74) (Figure 7D).

**Figure 8:**
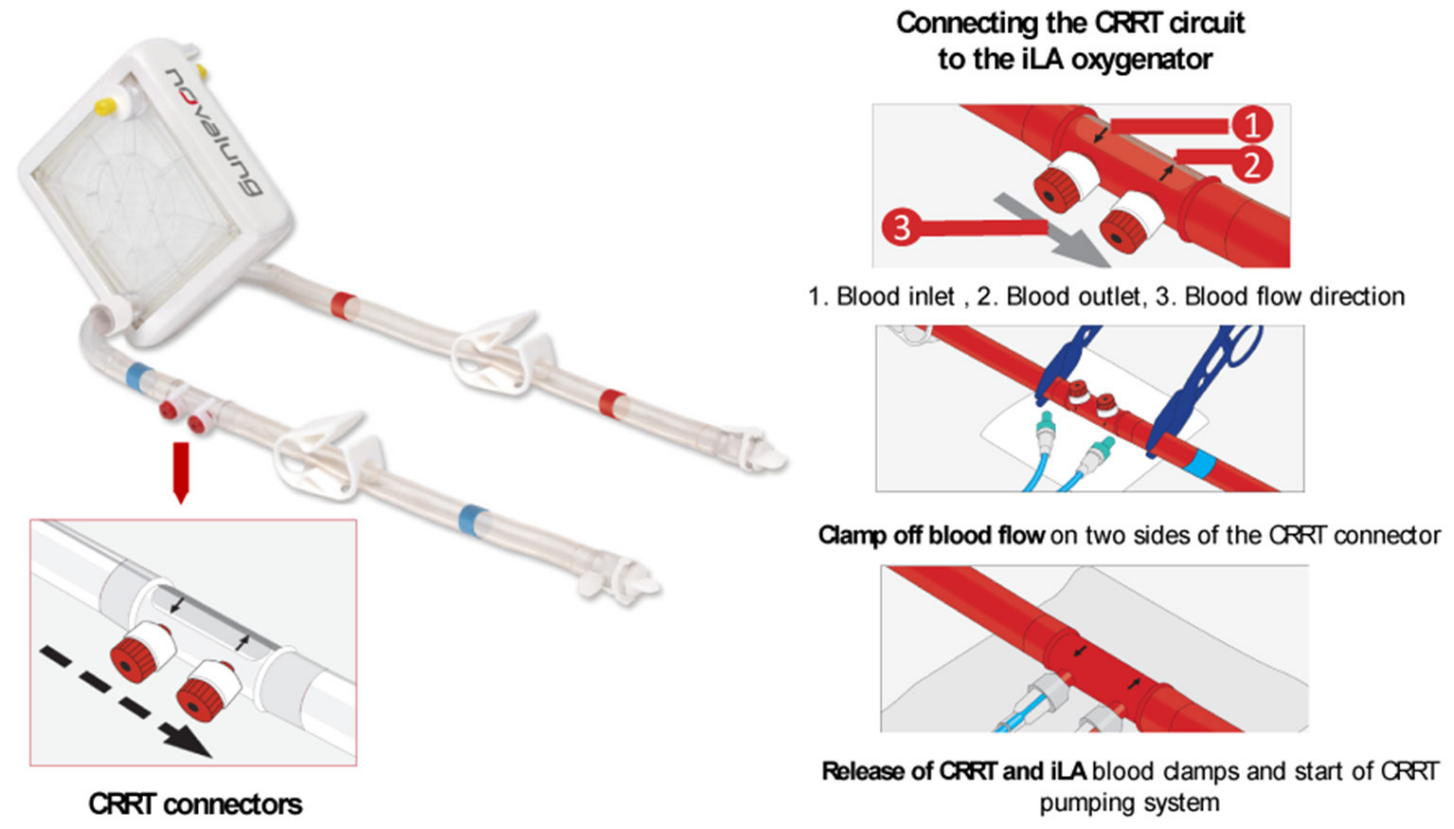
The iLA oxygenator (Xenios) offers CRRT connectors for simultaneous lungs and extracorporeal renal replacement therapy without the need of additional vascular access. The CRRT circuit is connected to the iLA system by the use of a inlet and outlet CRRT connector with the assistance of blood clamps. Modified from the ILA membrane ventilator (Xenios) procedure description (59).

ECMO systems have evolved to offer connectors between the gas exchange device and other organ support therapies. This is the case of the iLA oxygenators family of Xenios, which offers a CRRT connector for simultaneous lungs and extracorporeal renal replacement therapy (see Figure 8). The iLA oxygenator is connected to the patient *via* two femoral cannulas and no additional vascular access is required for the CRRT connection, which may reduce the risks of bleeding (75). Moreover, the membrane ventilator is suitable for connection with all types of CRRT techniques with blood flows up to 500 ml/min and can be used for 29 days. Seczyska et al. (12) describes this technique as simple and less invasive. Despite advantages in reducing the number of required vascular access for CRRT connection, available iLA connectors seem hemodynamically suboptimal as blood flows are deviated with a 90° angle to the CRRT access lines, see Figure 8, which can lead to disruption of blood flow, shear stress, and hemolysis. In addition, inlet and outlet blood flows are in close proximity, which could cause fluid turbulence and further shear stress. Moreover, the connection of the CRRT into the ECMO circuit requires the use of clamps to restrict blood flow while the CRRT circuit is being setup, which can also lead to hemolysis of red blood cells (13). In addition, each connection and disconnection of the CRRT machine to the ECMO circuit needs the assistance of a ECMO specialist/perfusionist and increases the risk of air embolism and clotting in both ECMO and CRRT circuits (71). It is important for blood flow to be restored quickly after the connection of the CRRT circuit to the ECMO device to avoid possible thrombosis risks.

## Technical Outlook

**Figure 9:**
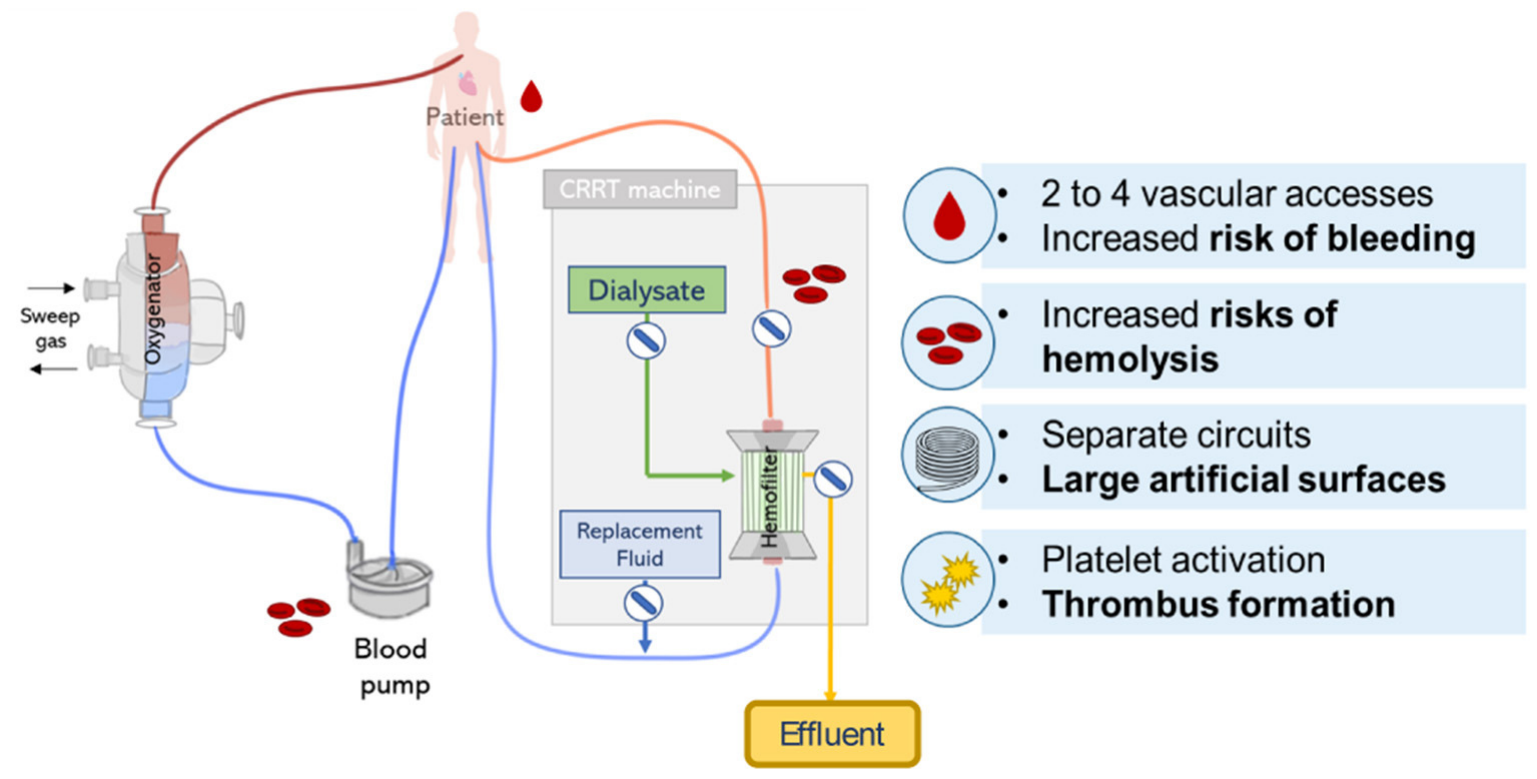
Lung and kidney support provided by parallel CRRT and ECMO circuits. Scheme represents possible circuit derived complications including risks of hemolysis, bleeding, and thrombus formation.

The combination of CRRT during ECMO can be lifesaving, however, it clearly increases the complexity of the treatment with potential additional risks. Recommendation on the safest and most effective approach of combining ECMO and CRRT is not clear and the decision typically depends on local clinical expertise, technical and medical availability (13). Despite the frequent combination of ECMO and CRRT treatment in ICUs, its implementation face multiple challenges related to the circuit complexity (12) leading to an increase in technical load, health care costs and patient management, and circuit related complications including bleeding, hemolysis, thrombus formation, and management of intra-circuit pressures (see Figure 9) (18).

### Circuit Complexity

Every component added to the ECMO circuit increases its complexity and may result in additional risks of infection, flow disturbances, and hemolysis (76) (see Table 2). Connecting an in-line hemofilter to the ECMO circuit requires the use of two external pumps to control the amount of fluid delivered to the patient, which besides increasing complexity, requires special attention from medical staff. Despite the frequent combination of ECMO and CRRT in an integrated approach, this technique requires the use of two blood pumps to circulate blood across the oxygenator and the CRRT machine, besides longer tubing lines, which are required to connect both circuits, rising the risks of hemolysis, heat loss, and increasing priming volume (11). Moreover, when CRRT and ECMO are connected in parallel, an additional large venous catheter must be introduced in a patient that already receives higher doses of anticoagulants, leading to severe risks of bleeding (75). Furthermore, an additional cannula is required by the CRRT connection to ensure sufficient blood supply. This could be an issue if higher ECMO output is required, since vascular access sites are occupied (12). Moreover, additional cannulation increases the risks of vascular and infectious complications (77).

**Table 2:** Comparison of number or access sites, number of pumps, and priming volume required for different ECMO and CRRT circuit connections.

**Circuit**	**Access cannulas**	**Number of pumps**	**Priming volume**
VV-ECMO (typical circuit)	1–2 access sites	1 ECMO roller or centrifugal blood pump	+
ECMO and in-line hemofilter	1–2 access sites	2 external pumps to control fluid balance	++
		1 ECMO blood pump	
CRRT Integrated in ECMO circuit	1–2 access sites	3 internal pumps to control fluid balance	++
		1 internal CRRT blood pump	
		1 ECMO blood pump	
ECMO and CRRT in parallel	2–4 access sites	3 internal pumps to control fluid balance	+ + +
		1 internal CRRT blood pump	
		1 ECMO blood pump	

*The sign (+) is used as a scale to represent circuit priming volume from lower priming volumes (+) to higher priming volumes (+++)*.

### Circuit Derived Complications

#### Artificial Surfaces

Artificial surfaces are used in the ECMO and CRRT circuit to carry blood and when the two circuits are combined additional tubing and circuit components increase the artificial surface to which the blood is into constant contact. The contact of blood with non-biological artificial materials leads to the activation of the coagulation system, platelets, and complement, which may result in formation of thrombus and device failure (78). Strategies to reduce such risks involve reducing the amount of artificial surfaces, using systemic inhibitors of coagulation. Modern ECMO devices are coated with heparin or other molecules to reduce coagulation activation. Despite improved hemocompatibility, the deposition of proteins onto artificial surfaces is still a challenge (1, 24, 79). This includes the formation of a limiting diffusion layer composed of fibrin, single cells, and cell clusters, which can cover large areas of the membrane surface, resulting in reduced gas transfer efficiency, lowered membrane function, and ultimately device failure (80). Other limiting factors relate to platelet and coagulation activation, resulting in clot formation within the system (79). These limitations are a major challenge for the long-term application of ECMO and the development of an implantable artificial lung (81). So far, commercial ECMO and CRRT devices can only be used for a short period of time. Therefore, it is primordial that future advances in the research field focus on the development of better anticoagulant molecules and ECLS circuits that better mimic the physiologic conditions for extended ECLS use (79). Moreover, research in new ECLS bio-hybrid materials with the use of an endothelization technique is an important step toward the creation of complete biocompatible materials (79, 82–86).

#### Hemolysis

Hemolysis can be defined as the premature disruption of red blood cells, which occurs outside of the circulatory system or within the circulatory system (87). Hemolysis is an intrinsic complication of extracorporeal support circuits that is most commonly caused by the mechanical stress applied to erythrocytes within the ECLS circulation. According to the Extracorporeal Life Support organization, hemolysis is characterized by levels of plasma free hemoglobin (PfHb) higher than 50 mg/dl and decreased levels of haptoglobin. Increased levels of PfHb have been related to higher morbidity, mortality (88), renal deficiency and are associated with an increased risk of thrombosis, pulmonary and systemic hypertension (89) and decreased organ perfusion.

During ECMO, hemolysis is mainly caused by excessive mechanical stress due to inadequate pressures (76), altered flows, and pump head thrombosis. Altered levels of PfHb caused by blood circulation in ECMO is potentially related to the development of hemolysis-induced AKI. Hemolysis was proven to be aggravated when CRRT is connected to the ECMO circuit (16). Costantini et al. demonstrated in a pioneer work that the presence of CRRT connected to the ECMO circuit was associated with both higher daily levels of plasma free hemoglobin and lower levels of haptoglobin. These parameters were selected as hemolysis markers and demonstrate the complex influence of kidney support and red blood cell trauma. The setup of the CRRT circuit can exacerbate hemolysis and lead to severe complications, including negative effects on multiorgan function. This is especially relevant considering that Costantini et al. (16) evaluated a CRRT integrated to the ECMO circuit, which is currently one of the most common connection modes used in ICUs.

#### Drug Dosage

ECMO patients are commonly treated with numerous drugs including sedatives, antibiotics, and anticoagulants. The absorption, distribution, and metabolism of drugs in ECMO patients can be affected by the interaction of these chemicals with elements of the ECMO circuit (90, 91). ECMO initiation requires the introduction of priming solutions, which may result in the dilution of plasma proteins and consequently affect the distribution of drugs that are protein-bound. Moreover, other elements of the ECMO circuit such as tubing and the oxygenator increase the surface area to which drugs can interact and adsorb (92). In addition, the deposition of proteins on the inner surface of the tubing and oxygenator membranes may increase sequestration of drugs with affinity to protein binding (93).

Lipophilic and highly protein bound drugs such as furosemide, warfarin or lorazepam are especially prone to sequestration in the ECMO circuit. Thus, sequestration of drugs by the ECMO circuit implicates changes in the choice and dosing of some drugs administered during ECMO (94). Similarly, drug metabolism in patients undergoing RRT can be affected by decreased kidney function and by the therapy itself. Reduced kidney function relates to an impaired glomerular filtration rate that particularly influences the elimination of hydrophilic drugs (95). Moreover, different RRT-specific factors including the mode of RRT, frequency of dialysis, and flow rates have an impact on drug removal (96). Additionally, filter type can influence drug clearance, since high permeability hemodiafilters lead to higher drug clearance rates than less permeable membranes (97). Therefore, clinicians must consider the complex drug interaction with both extracorporeal circuits in patients undergoing ECMO and CRRT when choosing doses for patients. One example of the combined effects of ECMO and RRT on the pharmacokinetics of an antibiotic drug was demonstrated by Shekar et al. While usual concentrations of the antibiotic (>2 mg/L) were maintained in all patients, higher doses (>8 mg/L) used for less susceptible microorganisms was achieved only in eight out of eleven ECMO patients, five of them on RRT.

#### Management of Intra-Circuit Pressures

Considering that the integration of a CRRT machine into the ECMO circuit is one of the most commonly used approaches for combined lung and kidney support (47), managing high CRRT pressures during ECMO is a major challenge (18). Few studies describe solutions to this issue. Rubin et al. (74) developed a method to manage pressures by connecting the inlet of a hemodialyzer to the arterial line of the ECMO circuit and its outlet to the venous line using three-way taps. However, since those connections are placed after the oxygenator, patients are exposed to risk of air embolism (18). Moreover, the use of three-way stop-cocks connected perpendicularly to the tubing may disturb blood flow and cause hemolysis. Santiago et al. (98) evaluated the introduction of a CRRT machine in the ECLS circuit after the centrifugal pump and before the oxygenator in pediatric patients. They opted for switching off CRRT pressure alarms. Nevertheless, the consequences of this practice have not been analyzed. The study of Tymowski et al. describes a stepwise method to manage high pressures in continuous veno-venous hemofiltration connected to ECMO. The method was efficient in avoiding iterative stops of the CRRT machines. However, the use of clamps to mitigate pressure in the return line of the circuit could cause blood damage. Therefore, the management of different working pressures in the ECMO and CRRT circuit is still a challenge. As a result, iterative stops of the CRRT machine resulting from iterative stops due to unsuitable pressure is one of the main factors decreasing CRRT efficacy (99). Besides iterative stops, membrane clotting is another main factor decreasing CRRT efficiency. The recommended blood flow rate of ECMO machines is between 2 and 5 L/min, which is 10 times higher than the typical blood flow rate for CRRT devices (0.15–0.2 L/min). This causes reduced lifetime for the hemo(dia)filtration membranes due to clotting (100). Avoiding membrane clotting is challenging, however guidelines indicate this can be achieved by ensuring blood flows between 150 and 300 ml/min (99). Thus, an important drawback relates to the fact that commercial ECMO and CRRT devices are set to work in different optimal conditions, which poses a challenge to the integration of their circuits.

### Rise in Technical Workload and Health Care Costs

A multidisciplinary clinical team composed of experienced surgeons, intensivists, nurses, clinical perfusionists, nutritionists and psychotherapists is required for the care of patients in combined ECMO and CRRT (101). Typically, a professional operator, an intensivist or nephrologist, is responsible for setting up the CRRT machine to the ECMO circuit (17). During operation, the management of combined therapies involves circuit controlling, monitoring of hemodynamics, anticoagulants, and respiratory parameters to prevent complications such as hemorrhage, hemolysis, infection and limb ischemia (102, 103).

Medical staff have to perform several tasks to manage the ECMO circuit and this workload is increased by the addition of a CRRT machine to the circuit (104). Nurses or clinical perfusionists caring for ECMO patients have to adjust pump flow according to hemodynamic parameters and cardiac function, manage the dose of anticoagulants that includes the monitoring of activated coagulation time every 2 h, manage mechanical ventilation, if necessary, monitor hemodynamic parameters by performing blood gas analysis and central venous oxygen saturation every 4 h, and manage the dose of vasoactive drugs (104). With the inclusion of a CRRT machine in the circuit, hemodiafiltration parameters have to be controlled. Therefore, nurses have to adjust the pump speed to control the delivery of replacement fluid and dialysate to the patient, adjust the potassium chloride dosage according to blood levels, and prime the CRRT tubing with 0.9% sodium chloride solution. The study of Ricci et al. evaluated nursing procedures during CRRT practices in Italy. Their analysis reports that nursing workload increased in 63% of the cases when CRRT had to be established in the ICUs, and that specific training was required for 50% of them to learn specific CRRT management (17). Therefore, in a similar way, the overall technical workload in ICUs could increase if CRRT needs to be added and managed within the ECMO circuit.

In consequence, the increase of technical workload would directly affect hospital costs of extracorporeal life support therapies. Lansink-Hartgring et al. reviewed the costs of ECMO treatment in hospitals across the world. ECMO stays and procedures costs up to US$ 318,187 per patient to American hospitals (105). Studies report that nursing days can account for 52% of total costs (14), and personnel costs correspond to the majority of additional costs in the ICU reaching values as high as US$ 67,000 (106). Therefore, an increase in nursing and technical work to monitor and manage both ECMO and CRRT circuits can have a great impact to hospital's budget. In addition, the cost of maintaining a renal support therapy during ECMO can sum up to 4% of the total costs of the ECLS therapy (14). The main factors contributing to CRRT costs include nursing staffing, dialysate and replacement fluid, anticoagulation and extracorporeal circuits costs, with a total ICU cost of US$ 3,629.80/day (15).

### Patient Management

ECMO patients stay an average of 52 days in the hospital, which are mainly constituted by days in the ICU (33 days) (106). In recent years, ECMO is generally established early in cases of ARDS to avoid possible complications related to mechanical ventilation, such as ventilatory induced lung injury. Some centers give preference to use ECMO as a first line of treatment, as alternative to mechanical ventilation, in awake non-intubated patients with respiratory failure. This technique is also known as “awake ECMO” and presents several advantages including reducing the amount of sedative drugs and delirium, facilitating patient rehabilitation, and allowing patients to communicate with relatives/friends and medical staff (102). Thus, reducing the amount of sedative drugs administered to the patient could reduce length of stay in the hospital. Considering that ECMO patients are critically ill and typically affected by muscle mass loss, they could benefit from rehabilitation sessions, reducing the incidence of neuromuscular disorders (107). Therefore, there is a strong rationale to believe that the use of VV-ECMO in awake patients could be applied in more clinical centers in the future. This would be especially relevant for patients waiting for a lung transplantation, considering their single organ disfunction and important benefit from preoperative physical rehabilitation (108).

The placement of a CRRT machine into the ECMO circuit challenges awake treatment, since it may require deeper sedation or protective restraints to reduce complications related to CRRT pressure alarms and prolong filter life (100). The use of sedative drugs increases complications and is related to longer stays in the hospital. Therefore, besides drawbacks in patient physical rehabilitation, prolonged length of stay of patients undergoing combined ECMO and CRRT increases health care costs. ECMO patients cost approximately US$ 5852 per day to a hospital (109), therefore, longer stays in clinics due to combined ECMO and CRRT treatment could substantially increase health care costs.

### Combined Pulmonary and Renal Support in a Novel Highly Integrated Artificial Device

Despite the frequent combination of ECMO and CRRT in ICUs, complications including increased risks of hemolysis, bleeding, and thrombus formation are important drawbacks of this method. Moreover, since lung and kidney support are provided in separate circuits, medical staff need to receive additional training and further control intra-circuit pressures to avoid disruptions during treatment. In addition, patients in combined ECMO and CRRT are generally under deeper sedation and stay longer in the hospitals, increasing the number of ICUs occupied beds, resulting in increased health care costs. Combined treatment using a CRRT and ECMO circuit is common, however, treatment seems to be far from optimal. There is a clear need for technologies that consider the cross talk between the lungs and the kidneys. Thus, researchers advocate the need for future extracorporeal support devices to achieve harmonization of components, techniques and function of multiple organ support therapies (110). For that, new highly integrated artificial lung technologies that combine functions for optimal multiple organ support, consider the relevant artificial organ cross talk, and avoid undesired side effects and operational drawbacks are desired (19, 110).

Specialists report the strong need for assimilating ECMO and CRRT treatments by possibly combining both techniques in a single extracorporeal circuit with integrated monitoring system (12, 19). This is in line with the trend of multipurpose extracorporeal support, where components, techniques, and operations are combined to avoid unwanted therapeutic side effects (110). Therefore, there is a strong rationale to believe future artificial lung technologies would be highly integrated. Furthermore, the cross-talk between the lungs and the kidneys is an important aspect to be considered in the creation of an implantable artificial lung. In this sense, combining lung and kidney support in one device exhibits relevant advantages. First, this approach would require only two vascular cannulae reducing the risk of bleeding. Second, combining both techniques in a single circuit would reduce the area of external artificial surfaces, reducing possible complication such as platelet activation, adhesion, aggregation leading to thrombus formation, and activation of the complement system (111). Furthermore, if a low-flow resistance system is developed, the created device could function even in the absence of a pump (112).

In a device combining alternative oxygenation and dialysis fiber layers inside the same housing, blood can flow around the fibers while oxygen and dialysate fluid can flow inside the fibers. The flow of dialysate through the fibers could be switched on and off depending on the patient's demand. Since blood is continuously flowing though both oxygenation and hemodiafiltration fibers, issues related to blood clotting are prevented when renal support is stopped. Thus this combined device would offer an additional possibility to modulate kidney support, which is so far not found in traditional configurations combining the ECMO and CRRT circuits.

**Figure 10:**
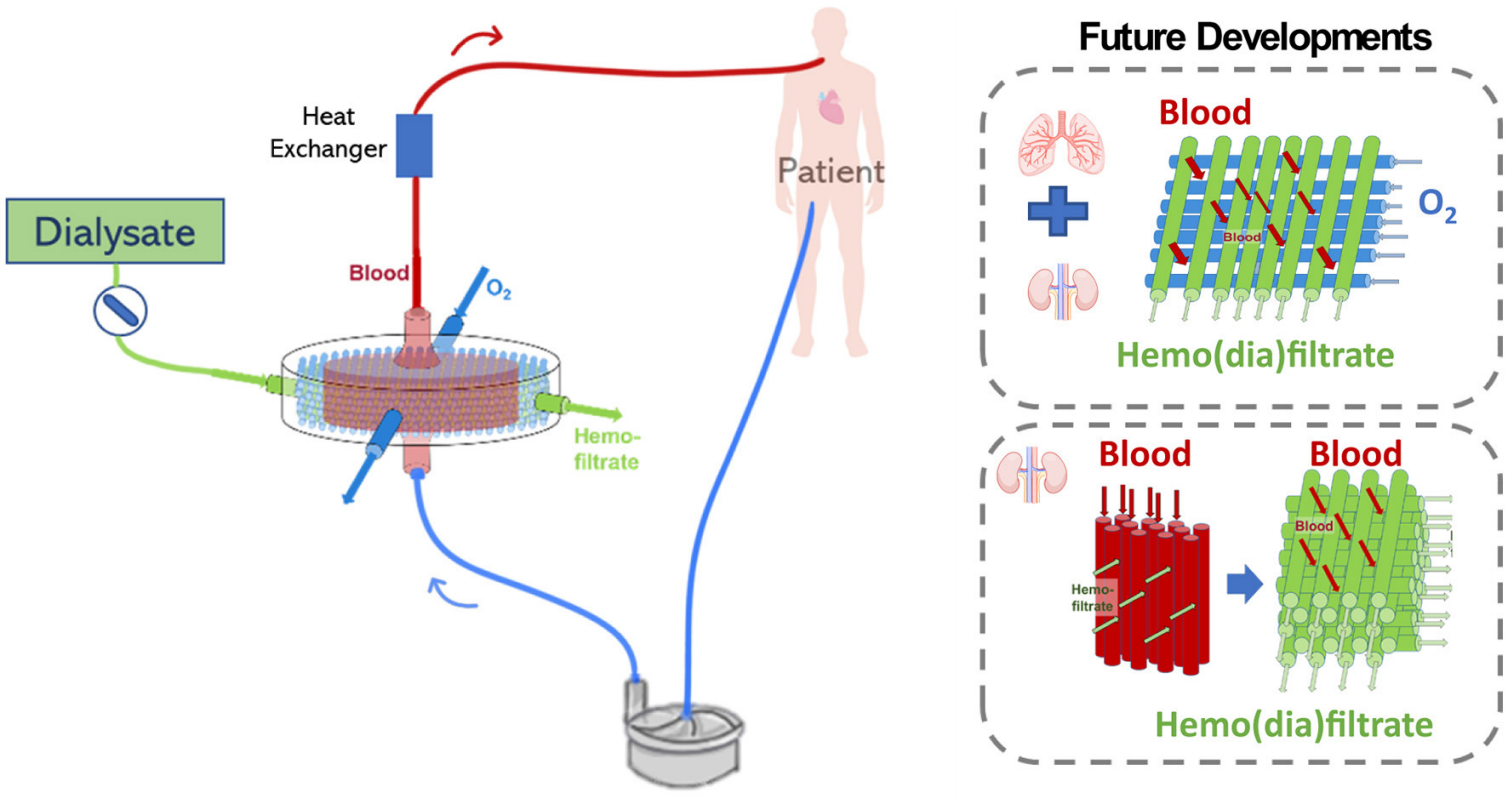
Possible challenges related to the development of an extracorporeal lung and kidney support device may include the configuration which oxygenation and hemofiltration fiber will be combined, the filtration mode, and future device miniaturization.

However, the combination of pulmonary and renal support in the same device would have to consider important points such as the combination of hemofiltration and oxygenation membranes, the type of filtration mode to modulate gas transfer and fluid balance in a sufficient rate, ultimately keeping blood oxygenation and fluid filtration in desired levels (112) (see Figure 10).

### Combined Lung and Kidney Support in a Single Extracorporeal Device

A modified Lung Assist (iLA) membrane ventilator combining oxygenation and hemofiltration membranes was developed by Wiegmann et al. The device was constructed by modifying the original fiber arrangement in the iLA device by alternatively placing 50% PMP gas exchange membranes and 50% polyether sulfone (PES) hemofiltration membranes. The study evaluated the efficiency of the device regarding its gas exchange and hemofiltration rate *in vitro* and *in vivo*. Results demonstrated that the modified fiber arrangement maintained an iLA-similar low flow resistance with no signs of hemolysis. Moreover, the innovative device presented stable *in-vitro* flow properties indicating the absence of thrombus formation, implying that it could work without a pump. In addition, gas transfer efficiency parameters demonstrated that the modified iLA had a stable gas exchange function with adequate oxygenation and decarboxylation over the duration of *in-vivo* experiments. This important work demonstrated for the first time the feasibility of combining pulmonary and renal support in a single device for the treatment of patients with severe lung and kidney injury. The design of the modified iLA device combining pulmonary and renal support was associated with several benefits. By combining PMP and PES membranes in the same housing, surface adverse effects such as inflammation and thrombus formation could be reduced. Moreover, for *in-vivo* application, only two large-caliber cannulas were necessary, also reducing the area of artificial surfaces to which blood is exposed to. Furthermore, potential risks associated with cannulation, including dislocation, bleeding and infection could be lowered (112). In addition, a different technology, the advanced organ support (ADVOS) system is reported to function as a renal replacement therapy with additional capacity to eliminate protein-bound substances and CO_2_ (113). The ADVOS is a multi-hemodialysis device based on albumin dialysis, which modulates the elimination of blood toxins into a dialysate by applying physicochemical changes such as pH to the dialysate fluid circulating in a secondary circuit. In this device, the elimination of CO_2_ occurs by playing with the equilibrium reaction between CO_2_, H^+^, and HCO${}_{3}^{-}$ as it would occur in the kidneys. ADVOS is operated in a range of blood flow rates (100–200 ml/min) much lower than ECMO (3–6 L/min), resulting in longer times between 2 and 4 h to normalize blood pH in patients. However, the ADVOS device does not provide complete lung support like in ECMO devices, since blood oxygenation is not supported, and CO_2_ elimination is limited. Furthermore, the combination of minimal-flow carbon dioxide removal (ECCO_2_R) systems and CRRT has been reported (114–116). Terragni et al. (114) and Moerer et al. (115) applied a CRRT-ECCO_2_R device [EQUA-smart®, Hemodec (MEDIA), Modena; Italy] to support mechanically ventilated patients. This combined system was found to facilitate lung protective ventilation. Nevertheless, minimal-flow ECCO2R systems have a limited capacity to eliminate CO_2_ and are insufficient to provide oxygenation for patients with severe respiratory insufficiency.

Therefore, to our knowledge, the modified blood oxygenator developed by Wiegmann et al. is the first prototype to offer complete lung and kidney support in one device. This device demonstrates that combining lung and kidney support is associated with important benefits, however, challenges including fiber arrangement, modulation of gas transfer and fluid filtration, device miniaturization, and precise anticoagulation regimen still have to be further addressed.

### Challenges in the Development of a Single Lung and Kidney Extracorporeal Support Device

#### Fiber Arrangement and Filtration Mode

Most available membrane lungs are constructed with multiple layers of microporous hollow fiber using the crossflow principle. In these devices, blood flows perpendicular to the fiber axis across the external surface of the fiber, while gas flows through the fiber lumen (2). When blood flows over the membrane bundle it generates mixing and transverse blood flow, which is essential for efficient gas exchange. The presence of diffusion boundary layers retards gas transfer, therefore, only the red blood cells that contact the vicinity of surface of each hollow fiber are oxygenated (117). Thus, blood mixing induced by the contact of blood with each successive fiber is essential to disrupt the boundary layer (2). Different studies found that the mass transfer performance of a membrane oxygenator depends on the arrangement of the hollow fiber membranes (58, 118). For this reason, commercial oxygenation membranes are produced in the form of knitted mats, which maintain each fiber at a predefined distance from each other in the same layer (56).

On the other hand, modern artificial kidney devices are composed of numerous semipermeable hollow fibers in a bundle. The semipermeable fibers act as a contactor between the blood and dialysate allowing solutes of different molecular weights to be transported by diffusion, convection, or adsorption across the membrane (62). These polymeric fibers predominately composed of synthetic PES or polysulfone (PSu) polymers are typically commercially available as individual fibers. Artificial kidney devices function in a “inside-out filtration” mode, meaning that blood flows through the lumen of the hollow fiber while dialysate flows outside-in the space between fiber (64). Therefore, opposed to what is found in membrane oxygenators, in blood filtrating devices, blood is contained inside the fiber. In this filtration mode, blood thrombi can be formed and deposited in the inlet of the fiber, therefore, long term treatment using hemo(dia)filtration fiber is limited due to fiber clogging and maximum filter life is typically <20 h (119).

Thus, from an engineering point of view, the configuration of fiber and filtration mode are therefore relevant points to be considered in the development of novel devices combining blood oxygenation and filtration membranes. Most probably, both hemofiltration and oxygenation fiber would need to be used in an “outside-in” approach, so blood could still flow outside of the fiber for efficient gas transfer. The concept of outside-in filtration for hemofiltration fiber has been tested by Dukhin et al. who evaluated the operation of a commercial dialyzers with blood flow outside the fiber. This study demonstrated for the first time that continuous and efficient hemofiltration was achieved for more than 100 h with outside-in operation (119). Therefore, the use of hemofiltration fibers combined with oxygenation fibers operating in outside-in mode could be feasible. However, for further implementation of this filtration mode, new hemo(dia)lfiltration fiber with a hemocompatible selective layer in the outer surface of the fiber would need to be developed. In addition, the configuration in which both types of fiber will be combined could affect device efficiency. Thus, as oxygenation fiber, hemofiltration membranes would have to be manufactured with a predefined interspacing between fibers. Moreover, the number of oxygenation and hemofiltration fibers inside the lung-kidney support device is a relevant parameter, since both efficient gas exchange and removal of blood toxins have to be achieved.

**Figure 11:**
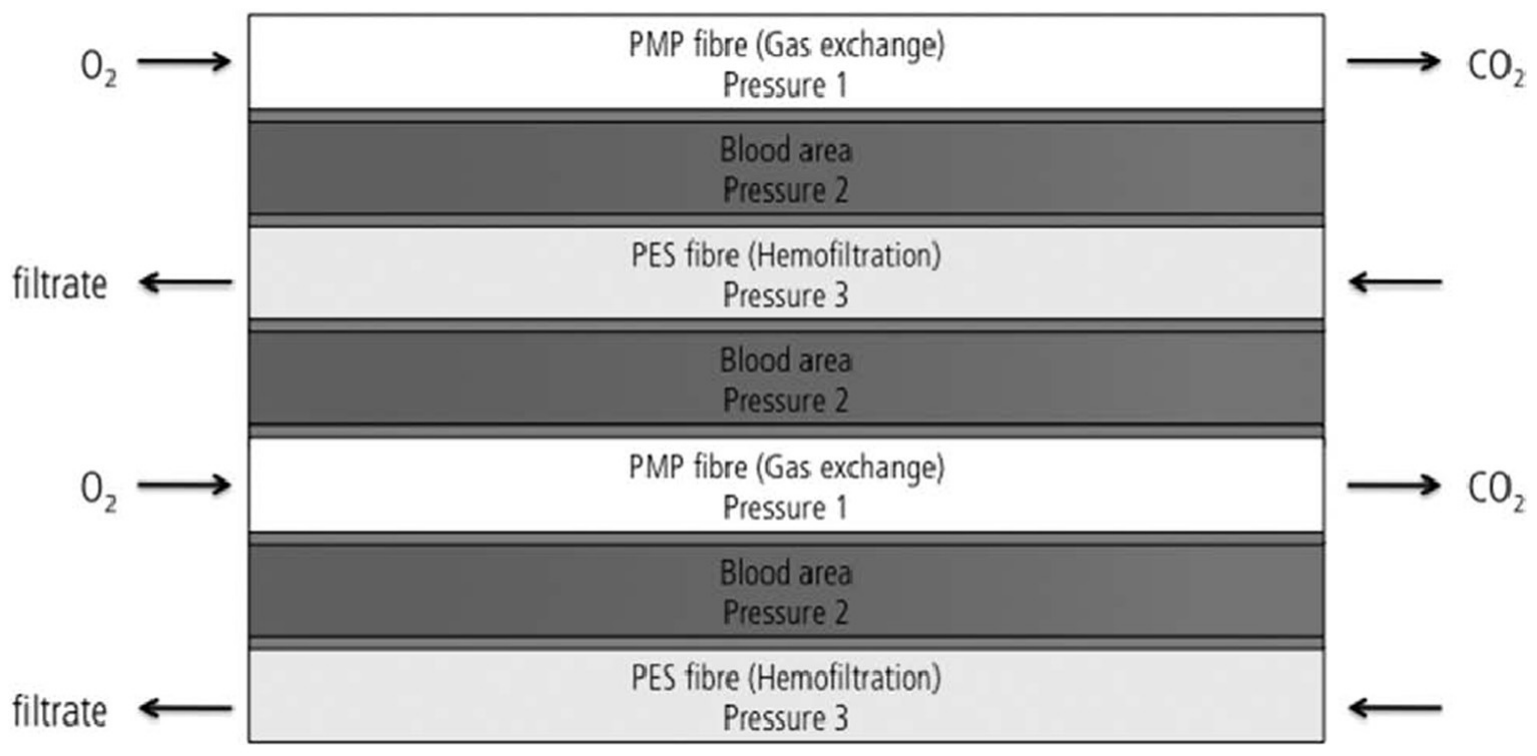
Design of the modified iLA membrane oxygenator combining 50% of oxygenation and 50% of hemofiltration fiber (112).

The modified iLA device developed by Wiegmann et al. provides an example of successful combination of oxygenation and hemofiltration fibers. It was constructed with a combination of 50% PMP gas exchange membranes and 50% hemofiltration membranes, placed as alternative and crossed right-angled layers inside the device (Figure 11). Blood flowed in one direction inside the device and a countercurrent exchange was achieved for both gas exchange and hemofiltration. However, this work does not provide specific details on how hemofiltration fiber-layers were arranged inside the novel device. Nevertheless, the selected fiber configuration and filtration mode was successful in achieving stable gas exchange and indicated a general feasibility of hemofiltration (112). However, further investigation of hemofiltration capacity in a system of three separate pressures (pressure in gas, blood and dialysate sections) is required.

#### Device Design

The design of an extracorporeal device combining lung and kidney support would have to translate the clinical needs of ECLS patients concerning their requirements on fluid management, kidney function and gas exchange. For that, a detailed analysis on the clinical needs of critically-ill patients such as requirements of fluid balance, elimination of creatinine and urea would have to be conducted to derive possible design specifications for the novel device. Furthermore, general requirements related to the development of an ECLS system would have to be considered, especially regarding a homogenous flow distribution, avoiding potentially critical zones regarding damage of blood cells, and optimized mass transfer. For this purpose, computational fluid dynamics (CFD) models could be used to evaluate and predict both stagnation and low flow areas and high shear areas in the novel ECLS device and facilitate device optimization (120). Furthermore, the future miniaturization of the artificial lung-kidney support device is an additional relevant challenge. This should consider how hemofiltration and hemofiltration fibers would be optimally combined, obtaining a balance between low surface area and volumes while keeping adequate gas and hemofiltration capacity (112).

#### Patient Management

The management of a combined lung and kidney extracorporeal support system could require a more precise anticoagulation regimen (48). Considering that the novel extracorporeal device combining lung and kidney support would function 24 h a day and 7 days a week to provide necessary oxygenation and CO_2_ removal rates for the patient, the use of hemofiltration fibers in these conditions would have to be studied. An important aspect regards filter survival times and possible anticoagulation regimes to avoid formation of blood clots in the circuit. Nowadays, clotting of the extracorporeal circuit is one of the most common complications during CRRT. Current practice in the use of anticoagulants during CRRT varies considerably (10), however, additional anticoagulation is usually not employed for patients undergoing conjunct CRRT and ECMO therapies. Studies report better filter survival times with the use of regional citrate anticoagulation for a conventional CRRT circuit compared to the use of unfractionated heparin (121). A different approach currently employed in clinics involves repeated application of heparin doses when a CRRT technique is started. Therefore, the administration of anticoagulation currently employed in ICUs during combined ECMO and CRRT treatments is already a complex task. Thus, maintaining therapeutic levels of anticoagulants with an extracorporeal device combining pulmonary and renal support could be comparable to the existing complexity in current technologies (112).

## Conclusions

Patients undergoing ECMO are at high risk of acute kidney injury and fluid overload. CRRT is the most commonly applied technique in ICUs to provide fluid management and kidney support for ECMO patients (11). CRRT and ECMO can be combined in multiple ways (12, 13). The integrated approach, which consists of connecting a CRRT machine to the ECMO circuit seems to be the preferred mode among centers (47). So far, all available modes for combining the ECMO and CRRT circuit present drawbacks related to increased complexity due to the use of separate circuits each requiring their own cannulas, pumps and tubing, circuit related complications such as increased risks of hemolysis, thrombus formation, and infection, as well as possible increase in technical workload and healthcare costs due to the management of ECMO and CRRT techniques. For this reason, a trend in extracorporeal lung and kidney support appears in the combination of ECMO and CRRT function in a single integrated extracorporeal device. The development of a novel extracorporeal device combining lung and kidney support seems feasible as demonstrated by the work of Wiegmann et al. However, the design of such device must consider relevant aspects such as the configuration which oxygenation and hemofiltration fiber will be combined, the use of outside-in filtration approach for hemofiltration fiber, the translation of patients' clinical needs and device miniaturization. These aspects are yet to be completely evaluated, however, it is clear that the development of a novel highly integrated artificial device combining lung and kidney support presents important advantages including decreasing the area of artificial surfaces and possible complications. In the future, a combined lung-kidney artificial support device could be used for the treatment of patients with severe lung diseases with or without restricted kidney function. Furthermore, novel designs expanding the concept of combined extracorporeal multiple organ support may potentially improve tailored therapy for individual critically-ill patients and improve intensive care therapies in innovative ways (112). In the future, it could be possible to tune how oxygenation and hemofiltration fibers with different specifications are combined in novel devices to offer a tailored treatment for specific patients or diseases modulating how molecules would be transferred and eliminated.

## Author Contributions

AM: investigation, conceptualization, writing—original draft, and visualization. FH: conceptualization, writing—review and editing, and supervision. BW and MN: conceptualization, writing—review and editing, and funding acquisition. JA: conceptualization, writing—review and editing, funding acquisition, and supervision. All authors contributed to the article and approved the submitted version.

## Funding

All authors are participants of the Priority Programme (Toward an Implantable Lung) (SPP 2014) funded by the German Research Foundation (DFG), Project No. 447746988.

## Conflict of Interest

The authors declare that the research was conducted in the absence of any commercial or financial relationships that could be construed as a potential conflict of interest.

## Publisher's Note

All claims expressed in this article are solely those of the authors and do not necessarily represent those of their affiliated organizations, or those of the publisher, the editors and the reviewers. Any product that may be evaluated in this article, or claim that may be made by its manufacturer, is not guaranteed or endorsed by the publisher.
